# Specific and Multi‐Product Clade I and Clade IV Sesquiterpene Synthases Contribute to the *Psilocybe cubensis* Volatilome

**DOI:** 10.1002/cbic.70318

**Published:** 2026-04-21

**Authors:** Sebastian Schober, Lisa Dorfmann, Karl Walther, Felix Blei, Andrew R. Chadeayne, Markus Gressler, Stefan Bartram, Sarah E. O’Connor, Dirk Hoffmeister

**Affiliations:** ^1^ Pharmaceutical Microbiology Friedrich‐Schiller‐Universität Jena Jena Germany; ^2^ Pharmaceutical Microbiology Leibniz Institute for Natural Product Research and Infection Biology ‐ Hans‐Knöll‐Institute Jena Germany; ^3^ CaaMTech, LLC Issaquah Washington USA; ^4^ Department Natural Product Biosynthesis Max Planck Institute for Chemical Ecology Jena Germany; ^5^ Cluster of Excellence Balance of the Microverse Friedrich‐Schiller‐Universität Jena Jena Germany

**Keywords:** chromatography, enzyme, *Psilocybe*, sesquiterpene, terpene synthase

## Abstract

Apart from the psychedelic psilocybin, the metabolite spectrum of *Psilocybe* “magic mushrooms” comprises sesquiterpenes, a class of natural products known to exhibit receptor‐modulating bioactivities. However, the composition of the sesquiterpene profile has largely remained an open question. Here, we report the characterization of five *Psilocybe cubensis* sesquiterpene synthases, both in vitro using recombinantly produced enzymes and in vivo in *Aspergillus niger*. CubF is a clade I α‐muurolol synthase. The investigated clade IV synthases were the near‐identical CubG1 and CubG2 synthases, which catalyze mainly *epi*‐isozizaene and β‐duprezianene formation. Furthermore, CubH and CubI were identified as primarily making dauca‐4(11),8‐diene and β‐barbatene, respectively. Gas chromatographic analyses of the headspaces of *P. cubensis* vegetative mycelium and fruiting bodies showed qualitative and quantitative differences, with sterpurene being among the major compounds in mycelium and dauca‐4(11),8‐diene in fruiting bodies. This fundamental knowledge of the *P. cubensis* terpenome may help distinguish the pharmacological effects of magic mushrooms versus pure psilocybin.

## Graphical abstract

Five sesquiterpene synthases of *Psilocybe cubensis* were investigated in vitro and in vivo. CubF is a clade I synthase producing α‐muurolol, whereas clade IV synthases CubG1 and CubG2 produce *epi*‐isozizaene and β‐duprezianene. Further clade IV synthases CubH and CubI primarily make dauca‐4(11),8‐diene and β‐barbatene, respectively. Our results help chart the natural product metabolome of one of the most iconic mushroom genera.

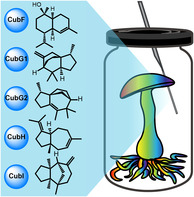

## 1 Introduction

The structural diversity of sesquiterpenes/‐terpenoids has intrigued generations of natural product chemists. Both plants and fungi show the capacity to morph the universal C_15_‐precursor (2*E*,6*E*)‐farnesyldiphosphate (FPP) into linear, mono‐ or oligocyclic and often chiral products, which become further functionalized in subsequent reactions [1]. For numerous sesquiterpenes/‐terpenoids, a biological activity has been demonstrated. Examples include the pharmaceutically used plant products α‐bisabolol used in topical treatments for its anti‐inflammatory properties [2], helenalin, which is an inhibitor of the transcription factor NF‐κB [3], or the antimalarial artemisinin [4]. Fungi, and mushrooms in particular, evolved a particularly diverse sesquiterpenoid metabolism [5]. The anticancer compound illudin S, made by the Jack O’Lantern mushroom *Omphalotus olearius*, and its semi‐synthetic derivative irofulvene are prominent examples [6]. Terpene synthases catalyze the key reaction, the cyclization of FPP to a cyclic product, which in some cases is preceded by isomerization to (3*R*)‐nerolidyldiphosphate (NPP). In some cases, only hydrolysis of FPP to an acyclic sesquiterpene is observed. Collectively, basidiomycete terpene synthases fall into four major evolutionary clades (I through IV), depending on the mode of cyclization and their substrate (FPP or NPP) [7, 8]. A fifth clade was proposed that also includes terpene synthases from termite mount‐inhabiting fungus‐garden‐forming mushrooms [8, 9, 10].

For decades, mushroom‐type fungi have been intensively analyzed for their sesquiterpenes/‐terpenoids [10, 11, 12, 13, 14]. However, the psychotropic *Psilocybe* magic mushrooms have not been considered for analysis of their terpenoid diversity for a surprisingly long time, even though a first insight dates back to 2009 with the discovery of psilosamuiensine, isolated from *Psilocybe* (*P*.) *samuiensis* [15]. Not until about 15 years later, a first exhaustive profiling of *Psilocybe* clade II and clade III synthases showed an unexpectedly diverse capacity of *P*. *cubensis* to produce sesqui‐ and even some monoterpenes [16, 17]. In addition to clade II and III synthases, members of all four major clades of terpene synthases are encoded in the genomes of *Psilocybe* species as well [18]. Most of these genes are actively transcribed [19] suggesting a diverse fungal (sesqui‐)terpenome whose constituent compounds remain only partially characterized. Nevertheless, secondary metabolites produced by *Psilocybe* species—including terpenes and terpenoids—are increasingly relevant for understanding the so‐called entourage effect. Within the context of *Psilocybe* mushrooms, this term describes the observation that equivalent amounts of psilocybin can produce distinct behavioral and pharmacological outcomes when administered as a pure compound versus as part of whole‐mushroom biomass or mushroom extracts [20]. Preclinical studies have demonstrated that *Psilocybe* mushroom extracts suppress marble‐burying behavior more effectively than authentic psilocybin at matched doses, without corresponding increases in locomotor impairment [21]. Toxicological studies further indicate that mushroom extracts can elicit stronger biological effects than isolated psilocin or psilocybin alone [22]. Together, these findings support the hypothesis that additional, as‐yet incompletely characterized fungal metabolites modulate the in vivo activity of psilocybin, motivating further investigation of the *Psilocybe* terpenome and related secondary metabolite pathways.

Continuing with *P. cubensis* as a model species, this study completes the survey on the *Psilocybe* sesquiterpenome by investigating selected clade I and IV synthases. We here report the in vitro and in vivo biochemical characterization of five sesquiterpene synthases. Among them was CubF, the first characterized *Psilocybe* clade I sesquiterpene synthase, which was identified as an α‐muurolol synthase. In addition, four multi‐product clade IV synthases were investigated, including CubG1, CubG2, CubH, and CubI.

## 2 Results and Discussion

### 2.1 Genetic Loci of Clade I and Clade IV Terpene Synthases

CubF‐like enzymes (>81% identical and >92% similar amino acids) are encoded in at least five *Psilocybe* species (*P. cubensis*, *P. cyanescens*, *P. serbica*, *P. mexicana*, *P. azurescens*, Figure S1, Table S1). In *P. cyanescens* and *P. azurescens*, the gene of one major facilitator superfamily (MFS)‐type transporter is located in the vicinity of *cubF* (two MFS transporter genes in other *Psilocybe* species). Other genes encoding standard natural product‐modifying enzymes were not found.

In contrast, the four sesquiterpene synthase genes (*cubG1*, *cubG2*, *cubH*, and *cubI*) appear specific to *P. cubensis*, as other *Psilocybe* species contain similar proteins of less than 70% amino acid identity. CubG1 and CubG2 are encoded in immediate vicinity in the genome, together with an internally truncated putative sesquiterpene synthase pseudogene (*cubG3*). The *cubH* gene is part of an extended cluster with genes for putative oxidoreductases and monooxygenases, and an additional but transcriptionally silent clade IV sesquiterpene synthase [19], and a potential Zn_2_Cys_6_ transcription factor. The *cubI* gene is embedded in a smaller cluster as well, comprising genes for three oxidoreductases and a decarboxylase (Figure S1). Hence, the CubH and CubI products may undergo further modification in *P. cubensis*.

### 2.2 Clade I Terpene Synthases

**FIGURE 1:**
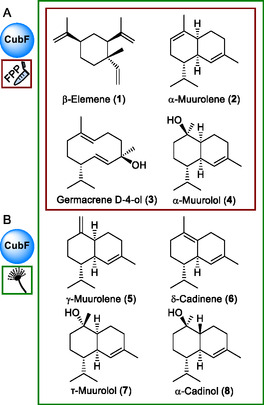
Chemical structures of *P. cubensis* terpene/terpenoid natural products, formed by CubF. (A) Products formed in vitro (red frame) using FPP as substrate; (B) Compounds additionally detected in extracts of *Aspergillus niger* tLD01, heterologously expressing *cubF* (green frame). Structures show relative configurations.

Clade I synthases catalyze an 1,10‐cyclization of (2*E*,6*E*)‐FPP, which proceeds through the *E*,*E*‐germacradienyl cation [5, 7]. *P. cubensis* encodes two clade I terpene synthases. Due to its conservation across multiple *Psilocybe* species and, thus, its general relevance, we chose CubF as our model. A phylogenetic analysis of its position relative to other basidiomycete clade I synthases (Figure S2, Table S2) placed CubF next to Cop3 and Agr3. The former is a *Coprinopsis cinerea* (gray shag mushroom) enzyme. Besides β‐elemene (**1,** Figure 1), Cop3 mainly produces α‐muurolene (**2**), along with germacrene D and other sesquiterpenes [23]. Likewise, Agr3 of the black poplar mushroom *Cyclocybe aegerita* catalyzes the formation of α‐muurolene (**2**) primarily as well, besides γ‐muurolene (**5**, Figure 1), δ‐cadinene (**6**), and α‐cadinol (**8**) [14]. Although the CubF cyclization mode was predictable and the phylogenetic placement unambiguous, we sought to experimentally identify CubF's actual product(s).

### 2.3 Characterization of CubF In Vitro and In Vivo

CubF is encoded by a 1489 bp gene including six introns, which leads to a 1059 bp reading frame in the mature mRNA and cDNA, respectively, encoding the 352 aa cyclase. We performed qRT‐PCR, which indicated constant expression of *cubF* both in fruiting bodies and in vegetative mycelium (Figure S3). The *cubF* cDNA was cloned to create the pET28a‐based expression plasmid pLD01, which encodes an N‐terminally hexahistidine‐tagged fusion enzyme. This plasmid was used to transform *Escherichia coli* KRX to produce a 42.2 kDa protein (Figure S4) in the induced culture.

**FIGURE 2:**
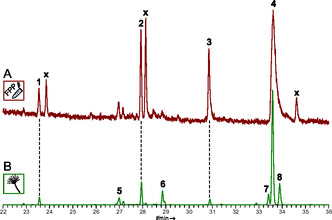
Gas chromatographic analysis of CubF activity assays. Chromatogram (A) Extract of the in vitro assay with FPP as substrate; (B) Mycelial extract of *A. niger* tLD01. Compound numbers: β‐elemene (**1**), α‐muurolene (**2**), germacrene D‐4‐ol (**3**), α‐muurolol (**4**), γ‐muurolene (**5**), δ‐cadinene (**6**), τ‐muurolol (=*epi*‐α‐muurolol, **7**), α‐cadinol (**8**). Impurities are indicated with (**x**).

CubF was purified by immobilized nickel affinity chromatography (IMAC) and incubated with FPP as substrate for in vitro product formation assays. After extraction with *n*‐hexane, gas chromatography, coupled to electron impact mass spectrometry (GC‐EIMS), revealed four sesquiterpene product peaks (Figure 2) which were compared with mass spectra and retention indices of databases for identification (Table S3) [24, 25, 26, 27]. The peaks were identified as β‐elemene (**1**), as α‐muurolene (**2**), as germacrene D‐4‐ol (**3**), and as α‐muurolol (=torreyol, **4**, Figure 1), which represented the major product (72.7% of total peak area).

To confirm these in vitro results independently and in a cellular environment, CubF was heterologously produced in *Aspergillus* (*A*.) *niger*, transformed with plasmid pLD06. The resulting transformant *A. niger* tLD01 carried the *cubF* cDNA integrated into the genome (Figure S5) and allowed for doxycycline‐dependent induction [28]. *n*‐Hexane mycelial extracts, harvested from induced versus non‐induced cultures of *A. niger* tLD01, were prepared for gas chromatographic and mass spectrometric (GC–MS) analysis.

Furthermore, we included extracts from (i) a control that carried the insertless vector (*A. niger* tPS01) [29] and (ii) from the untransformed parental strain *A. niger* ATNT16ΔpyrGx24 [28, 30] as the second control. (Figure 2, Figure S6). GC–MS analysis of the *A. niger* extracts confirmed the results from the in vitro assays (Table S4). Again, α‐muurolol (=torreyol, **4**) was the dominant product (57.8% of the total peak area). In addition, peaks were detected which were assigned to sesquiterpene/‐terpenoid compounds γ‐muurolene (**5**), δ‐cadinene (**6**), τ‐muurolol (=*epi*‐α‐muurolol, **7**), and α‐cadinol (**8**). These findings confirm earlier analytical work [17] in which α‐muurolene (**2**) and α‐muurolol (=torreyol, **4**) were the main terpenoid compounds of *P. cubensis* mycelium but add another six sesquiterpenes/‐terpenoids to the *Psilocybe* secondary metabolome that have not been reported yet to occur in this mushroom genus.

### 2.4 Clade IV Terpene Synthases

The second focus of this study were clade IV synthases. The products of this class of synthases have not been predicted or experimentally verified for the entire genus *Psilocybe*. Clade IV synthases typically catalyze the 1,6‐cyclization of (3*R*)‐NPP proceeding through the bisabolyl cation as central intermediate [31]. *P. cubensis* codes for a total of eight clade IV terpene synthases. We selected CubG1 and CubG2 as their genes are co‐localized (Figure S1), and their transcription is upregulated in fruiting bodies versus vegetative mycelium, albeit to a different extent (Figure S3). We also included CubH as its gene is located in a larger putative cluster of biosynthesis genes. Finally, CubI was chosen due to the regulation pattern of its gene *cubI*, with down regulation in fruiting bodies, compared to mycelium (Figure S3). The phylogenetic tree (Figure S2) of basidiomycete clade IV sesquiterpene synthases reveals that CubG1 and CubG2, like CubH and CubI, cluster with terpene synthases Tps1A and Tps2A, i.e., enzymes that catalyze *α*‐bisabolene formation in the Taiwanese medicinal mushroom *Taiwanofungus camphoratus* (syn*. Antrodia cinnamomea*) [32].

### 2.5 Characterization of CubG1 and CubG2 In Vitro and In Vivo

**FIGURE 3:**
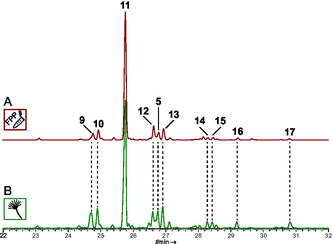
Gas chromatographic analysis of CubG1‐catalyzed sesquiterpene formation in vitro with FPP as substrate, or in vivo. Assays or mycelia were extracted with *n*‐hexane. (A) Extract of the in vitro assay and (B) extract of a doxycycline‐induced culture of *A. niger* tLD02. Chromatograms of controls are shown in Figure S7. Compound numbers: β‐cedrene (**9**), acora‐3,5‐diene (**10**), *epi*‐isozizaene (**11**), β‐acoradiene (**12**), γ‐muurolene (**5**), 10‐*epi*‐β‐acoradiene (**13**), β‐bisabolene (**14**), α‐alaskene (=γ‐acoradiene) (**15**) (*E*)‐γ‐bisabolene (**16**), unknown (**17**, 123, 222, RI 1575).

**FIGURE 4:**
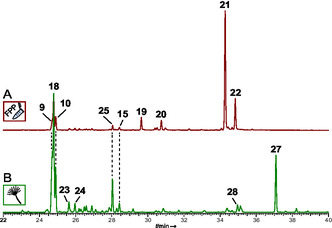
Gas chromatographic analysis of CubG2‐catalyzed sesquiterpene formation. (A) Extract of the in vitro assay with FPP as substrate and (B) *A. niger* tLD03 transformant extract after induction with doxycycline. Chromatograms of controls are shown in Figure S8. Compound numbers: β‐cedrene (**9**), acora‐3,5‐diene (**10**), α‐alaskene (=γ‐acoradiene) (**15**), β‐duprezianene (**18**), unknown (**19**, 119, 204, RI 1543, base peak, molecular ion, retention index), unknown (**20**, 119, 204, RI 1572), acorenol (**21**), unknown (**22**, 119, 222, RI 1681), sesquisabinene A (**23**), and amorpha‐4,11‐diene (**24**), and (*Z*)‐α‐bisabolene (**25**), and unknown (**26**, **27**).

**FIGURE 5:**
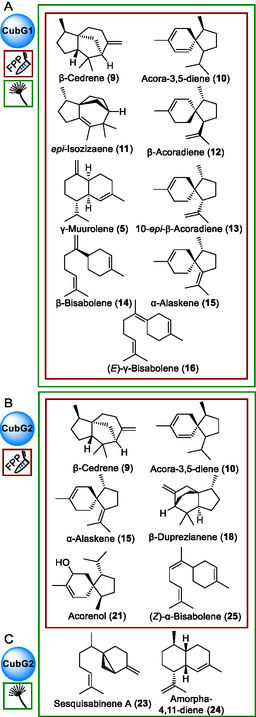
Chemical structures of *P. cubensis* sesquiterpene/‐terpenoid natural products. (A) Products of CubG1, formed in vitro with FPP as substrate and compounds additionally detected in the *cubG1*‐expressing *A. niger* tLD02; (B) compounds of CubG2 terpenoid formation in vitro with FPP as substrate and (C) additional compounds detected in extracts of *A. niger* tLD03. Panels A–C show relative configurations.

These sesquiterpene synthases are encoded by two paralogous genes of 1227 bp (*cubG1*) and 1218 bp (*cubG2*), each including four introns. The cDNAs (981 bp and 990 bp) lead to enzymes of 326 aa and 329 aa (pairwise sequence identity is 72.0%, calculated molecular masses: 37.6 and 37.9 kDa). Following the RT‐PCR, the *cubG1* and *cubG2* cDNAs were separately inserted into vector pET28a to create expression plasmids pLD02 and pLD03. They were used to transform *E. coli* KRX and to produce and purify CubG1 and CubG2 as N‐terminally hexahistidine‐tagged enzymes (40.1 and 40.2 kDa) in the two induced cultures (Figure S4). After purification by IMAC, CubG1 and CubG2 were investigated in in vitro product formation assays with FPP as substrate. As for CubF, the CubG1 and CubG2 assays were extracted with *n*‐hexane, analyzed by GC–MS, and the mass spectra and retention indices compared with databases [24, 25, 26, 27], with pure compounds, and with plant essential oils (Cubeb, Norwegian Angelica, Hinoki Cypress, Red Cedar, and Key Lime). CubG1 and CubG2 are multi‐product synthases (Figure 3, Figures S7 and S8, Tables S5–S6). With 67.4% of the total peak area, the major product of CubG1 in vitro assay was *epi*‐isozizaene (**11**, Figures 4, 5).

Other products that each contributed >2% to the total peak area were β‐cedrene (**9**, 4.3% peak area), and acora‐3,5‐diene (**10**, 4.3% peak area). Very minor amounts of further sesquiterpenoids were present as well. Products resulting from in vitro CubG2 assays indicated a partially divergent spectrum of compounds compared to CubG1. Identified sesquiterpenoids were β‐cedrene (**9**, 4.3% peak area) and acora‐3,5‐diene (**10**, 5% peak area), both of which were found in very similar proportions in CubG1 assays as well. However, unlike with CubG1, the major compounds included acorenol (**21**, 46.3%, Figure 5) and β‐duprezianene (**18**, 13.9% peak area), along with three putative sesquiterpenes that remained unidentified (Table S6).

We verified the results of the in vitro assays by independent in vivo work. *A. niger* was transformed with plasmids pLD07 or pLD08 to inducibly, but separately, express cDNAs of *cubG1* and *cubG2* after integration of the genes into the host's genomic DNA (Figure S5). The terpene product spectra of these transformants, *A. niger* tLD02 and tLD03, were chromatographically investigated for sesquiterpene products (Figure 4, Figures S7 and S8, Tables S7 and S8). As for CubF, three controls were run in parallel, each including uninduced cultures of *A. niger* tLD02 and tLD03, respectively, as well as *A. niger* tPS01 and the untransformed host strain as background controls. The sesquiterpene profile of CubG1, found in vivo, matched that of the in vitro assay. The dominant product was again *epi*‐isozizaene (**11**, 53.3% of the total peak area), followed by β‐cedrene (**9**, 8.0%) and various other minor compounds.

In contrast, the in vitro and in vivo sesquiterpene profiles of CubG2 diverge somewhat, as acorenol (**21**), the major product found in vitro, was not detected in vivo; only its direct precursor, acora‐3,5‐diene (**10**, 11.4% of the total peak area), was present. β‐duprezianene (**18**) was the most abundant product (26.6%, Table S8), followed by β‐cedrene (**9**, 18.7%), both of which were also found in vitro. A putative sesquiterpene (15.0% of the total peak area) that remained unidentified was the third most abundant product of CubG2 in vivo. Further products were identified (Figure 4), among them (*Z*)‐α‐bisabolene (**25**, 6.9%).

### 2.6 Characterization of CubH In Vitro and In Vivo

Next, we characterized CubH whose gene is transcribed nearly equally in fruiting bodies and vegetative mycelium (Figure S3). The chromosomal gene of 1195 bp, including six introns, corresponds to a 951 bp reading frame and encodes a native protein of 316 aa with a calculated molecular mass of 36.3 kDa.

As with the previous terpene synthases, the enzyme was produced recombinantly by an induced culture of *E. coli*, this time transformed with the expression plasmid pLD04 (Figure S5) which led to a 38.6 kDa N‐terminally hexahistidin‐tagged enzyme (Figure S4).

**FIGURE 6:**
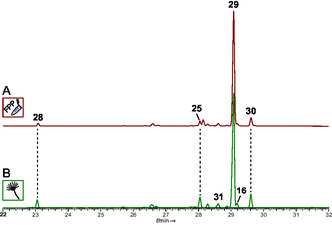
Gas chromatographic analysis of terpene formation by CubH. (A) in vitro product formation assay with CubH and FPP; (B) doxycycline‐induced culture of *A. niger* tLD04. Compound numbers are: (*E*)‐γ‐bisabolene (**16**), (*Z*)‐α‐bisabolene (**25**), daucene (**28**), dauca‐4(11),8‐diene (**29**), unknown (**30**, 93, 204, RI 1544), unknown (**31**, 119, 204, RI 1518).

**FIGURE 7:**
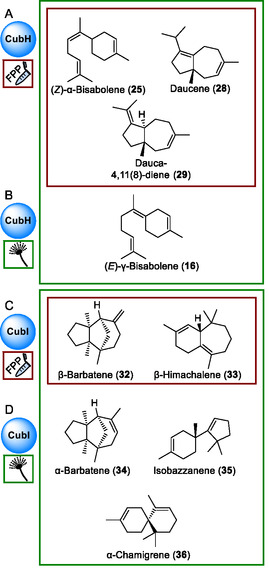
Chemical structures of *P*. *cubensis* terpene/terpenoid natural products. (A) Products of CubH, formed in vitro (red frame) with FPP as substrate and (B) additional compounds found in the *cubH*‐expressing host *A*. *niger* tLD04 (green frame). (C) terpenoid formation by CubI in vitro with FPP as substrate (red frame). and (D) further compounds in extracts of *cubI*‐expressing *A*. *niger* tLD05 (green frame). Structures show relative configurations.

CubH was tested for product formation in vitro by incubating with FPP. Subsequent GC‐EIMS analysis showed several product peaks (Figure 6) which were identified by comparison of mass spectra and retention indices, as before (Table S9) [24, 25, 26, 27]. The principal product was dauca‐4(11),8‐diene (**29**, Figure 7, 83.1% of the total peak area), along with an unidentified metabolite (**30**, peak area 6.3%, 93, 204, RI 1544, base peak, molecular ion, retention index) and two other minor products >2% of the peak area, including (*Z*)‐α‐bisabolene (**25**, 3.1% peak area) and daucene (**28,** 2.1% peak area).

In parallel, CubH was also produced in *A. niger* tLD04 with plasmid pLD09 integrated into its genome (Figure S5). Dauca‐4(11),8‐diene (**29**) was again the predominant product (72.8% of the total peak area, Table S10), minor compounds were (*Z*)‐α‐bisabolene (**25**), daucene (**28**), and an unknown compound (**30**). Gas chromatographic analysis confirmed the findings made for the in vitro assays. However, another bisabolene, (*E*)‐γ‐bisabolene (**16**), was found in vivo with a peak area of 2.3%, respectively, whereas this compound was formed merely in traces in vitro.

### 2.7 Characterization of CubI In Vitro and In Vivo

**FIGURE 8:**
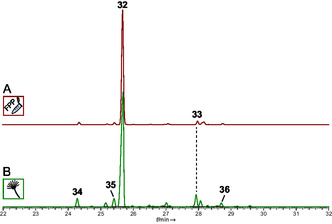
Gas chromatographic analysis of extracts of terpene formation by CubI. (A) In vitro assay with FPP as substrate (B) extract of *A. niger* transformant tLD05 Compound numbers: β‐barbatene (**32**), β‐himachalene (**33**), α‐barbatene (**34**), isobazzanene (**35**), α‐chamigrene (**36**).

We concluded our investigation of *P. cubensis* clade IV sesquiterpene synthases by characterizing CubI. Its gene *cubI* comprises 1205 bp of gDNA including four introns and a 972 bp reading frame, respectively. The CubI enzyme is 323 aa long and has a calculated molecular mass of 36.7 kDa. The *cubI* cDNA was ligated to pET28a, to yield plasmid pLD05 to produce a N‐terminally hexahistidine‐tagged CubI version in *E. coli* KRX. Following metal affinity chromatography (Figure S4), the pure 39.0 kDa fusion protein was assayed whether it would catalyze sesquiterpene formation in vitro in the presence of FPP. The gas chromatographic analysis showed a near‐exclusive formation of one compound that was identified as β‐barbatene (**32**, Figures 7 and 8, 90.9% of the total peak area), along with very minor amounts of β‐himachalene (**33**, 2.5% peak area) as second product. These findings were confirmed by independent in vivo work using *A. niger* tLD05 which carries plasmid pLD10 integrated in its genome to express the *cubI* cDNA (Figure S5). Like in the in vitro assay, the sesquiterpene product spectrum of this transformant included β‐barbatene (**32**) as clearly dominant compound, accompanied by β‐himachalene (**33**) (76.7% and 5.5% peak area, respectively, Figure 8, Figure S10, Tables S11 and S12). Additional minor products with a peak area >2% were α‐barbatene (**34**, Figure 7), isobazzanene (**35**), and α‐chamigrene (**36**) with peak areas of 3.9, 4.1, and 2.5% of the total area, respectively.

Our results on *P. cubensis* clade IV sesquiterpene synthases are congruent with earlier findings on (*Z*)‐*α*‐bisabolene (**25**)‐forming *Taiwanofungus camphoratus* synthases Tps1A and Tps2A [32] that show close phylogenetic relationship (Figure S2). Bisabolenes were verified as minor products of CubG1, CubG2, and CubH as well. Furthermore, the more distantly related *Omphalotus olearius* sesquiterpene synthase Omp9 and Omp10 catalyze formation of α‐ and β‐barbatene (**34**, **32**), and of daucene (**28**) and dauca‐4(11),8‐diene (**29**), respectively [10]. Hence, these enzymes possess a metabolic capacity comparable to CubI and CubH. Still, mushroom terpene synthases such as CubG1 and CubG2 which catalyze mainly *epi*‐isozizaene (**11**) and β‐duprezianene (**18**), have not been described yet.

### 2.8 Headspace Analysis of Mycelium and Fruiting Bodies

Previous analytical work had primarily focused on in vitro assays or heterologous systems. Motivated by earlier work on volatiles emitted by the *Omphalotus olearius* (the Jack O’ Lantern mushroom) [10] and *Cyclocybe aegerita* (Poplar mushroom) [33], we collected information on sesquiterpenes released by both *P. cubensis* vegetative mycelium and fruiting bodies. Furthermore, mycelial damage, caused by predators or microbial cell wall‐degrading enzymes, induces natural product formation [34, 35, 36, 37]. Therefore, in a set of parallel experiments, we treated the fungal material with lysing enzymes. This first headspace analysis of a *Psilocybe* species was carried out by solid phase micro extraction fibers placed above slant cultures or mature fruiting bodies.

**FIGURE 9:**
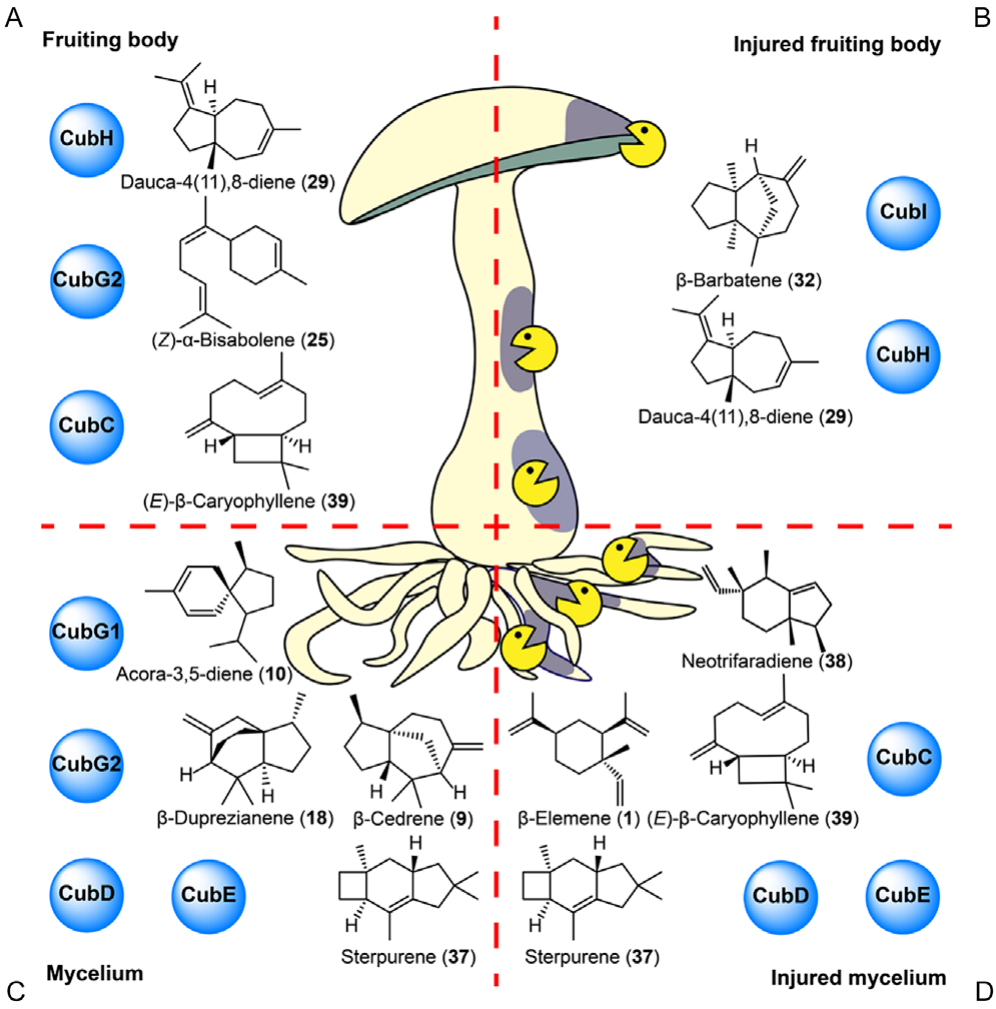
Chemical structures of the major volatile sesquiterpenoids identified by SPME headspace analysis. (A) *P. cubensis* fruiting bodies, (B) fruiting bodies treated with lysing enzymes, (C) untreated mycelium, and (D) mycelium treated with lysing enzymes. All structures show relative stereochemistry.

In the headspace of untreated mycelium, sterpurene (**37**), a product of sesquiterpene synthases CubD and CubE [17], was found (Figure 9, Table S13), along with CubG1/CubG2 products β‐cedrene (**9**), acora‐3,5‐diene (**10**), and β‐duprezianene (**18**). Mycelium injured with lysing enzymes showed a changed set of sesquiterpenes, with sterpurene (**37**) being the major product, whereas β‐cedrene (**9**) and β‐duprezianene (**18**) were only present in traces. However, neotrifaradiene (**38**), whose biosynthetic enzyme is not known in *P. cubensis* so far, was detected as second main product, along with various minor products (Figure 9, Table S13).

In the headspace of untreated fruiting bodies, we identified the CubH product dauca‐4(11),8‐diene (**29**), and the CubG2 product (*Z*)‐α‐bisabolene (**25**) as main components (58.0 and 14.8% of the total peak area). As with vegetative mycelium, cell wall damage changed the sesquiterpene composition in the headspace of mushroom fruiting bodies as well. While dauca‐4(11),8‐diene was also present with treated mushrooms (29.4%), (*Z*)‐α‐bisabolene (**25**) was not detected, while β‐barbatene (**32**) was quantitatively dominant (70.6%).

## 3 Conclusion

Combined with previous research [16, 17], this work completes a comprehensive survey on the biosynthetic capacity of *P*. *cubensis* to form sesquiterpenes/‐terpenoids. This species encodes active sesquiterpene synthases of all four major clades (I–IV). Some synthases are encoded in other *Psilocybe* species, whereas some seem species‐specific. Therefore, we expect qualitative differences in the sesquiterpene repertoire among the species.

Our results shed light on the non‐alkaloid secondary metabolome of psychedelic mushrooms. We conclude that our data may help explain the chemical basis of entourage effect and, more generally, address the intriguing but still open question to what extent the—perhaps species‐specific—pharmacological effects of psilocybin‐producing mushrooms on the human body differ from those exerted by pure psilocybin.

## 4 Experimental Section

### 4.1 Materials and Microorganisms

Chemicals, salts, media ingredients, and organic solvents were procured from Carl Roth, Merck, Sigma–Aldrich, and VWR. Essential oil references were from Oshadhi GmbH, Bühl, Germany. Oligonucleotides were custom‐synthesized by IDT Europe. *Escherichia coli* KRX (Promega) was the heterologous host to produce enzymes*. A. niger* ATNT16Δ*pyrG*x24 [28] was used for terpene/terpenoid formation in vivo. The strains were grown in *Aspergillus* minimal medium (AMM) with 100 mm d‐glucose and 20 mm l‐glutamine as carbon‐ and nitrogen sources, respectively, in shake flask cultures kept at 140 rpm and 30°C. For natural product and qRT‐PCR analyses, *P. cubensis* strain FSU12409 [38] was used. Fruiting bodies of *P. cubensis* were produced as previously described [39]. Fungal biomass was produced on solid malt extract peptone (MEP) medium (15 g L^−1^ malt extract, 3 g L^−1^ peptone, 18 g L^−1^ agar). The cultures were incubated for 7–9 days at 25°C.

### 4.2 Phylogenetic Analyses

The phylogeny of clade I and IV sesquiterpene synthases and the phylogenetic position of synthases CubF‐CubI among other basidiomycete synthases were inferred using the IQTree Web Server [40]. Initially, the sequences of clade I–V synthases were aligned using ClustalW2 [41], as part of the Geneious software package, version 10.2.4. Please see Table S2 for details on database entries for clade I and IV synthases. The alignment was processed by the IQTree tool in ultrafast bootstrap (UFBoot) mode [42] (VT + F+G4 best‐fit model, with 1000 replicates). Furthermore, accuracy was checked by the SH‐aLRT test [43]. Bootstrap values are given at each node in case the SH‐aLRT was >80%.

### 4.3 qRT‐PCR Analysis of Terpene Synthase Gene Transcripts

*P. cubensis* mycelium and fruiting bodies were quick‐frozen in liquid nitrogen and ground to a powder, using mortar and pestle. RNA was isolated with the SV Total RNA Isolation kit (Promega). To remove residual DNA, the isolated RNA was treated with Baseline‐Zero (Lucigen). Chromosomal DNA was isolated as described [44] and subsequently used to determine the efficiencies of oligonucleotide primers. cDNA necessary to analyze gene expression and to clone the synthase genes was produced by reverse transcription (1 µg RNA template) with the Thermo RevertAid RT kit and an oligo‐(dT)_18_ primer. The qRT‐PCRs were set up in a total volume of 20 µL and included 300 ng DNA template, 2 µm oligonucleotide primer (each) and the EvaGreen qPCR mix (Bio&Sell). Sequences, target genes, and oligonucleotides efficiencies are shown in Table S15. PCR parameters were as follows: 95°C/15 min hold, 40 cycles of denaturation at 95°C/15 s, annealing at 60°C/20 s, extension at 72°C/20 s. Three biological and three technical replicates were performed. The glyceraldehyde‐3‐phosphate dehydrogenase (*gpdA*) gene as internal housekeeping reference, and an established carpophore‐specific marker gene (*mtdA*) [45] served as controls. Gene expression levels were determined as described [46].

### 4.4 Construction of Expression Plasmids

To produce terpene synthases in *E. coli*, expression plasmids pLD01, pLD02, pLD03, pLD04, and pLD05 were constructed, using vector pET28a in which *cubF*, *cubG1*, *cubG2*, *cubH*, and *cubI*, respectively, were integrated. The inserts were PCR‐amplified in a volume of 50 µL and in the appropriate buffer. The reactions contained 2 µm MgCl_2_, 200 µm each dNTP, 10 µm each oligonucleotide primer, cDNA from the previous qRT‐PCR as template, and 1 U Phusion HF DNA Polymerase. Oligonucleotide primer details are given in Table S16. Primers were oLD01/oLD02 to amplify *cubF*, oLD04/oLD05 for *cubG1*, oLD07/oLD08 for *cubG2*, oLD10/oLD11 for *cubH*, and oLD13/oLD12 for *cubI*. Thermal cycling was: 95°C/2 min hold, 31 cycles of denaturation at 95°C/20 s, annealing at 60°C/20 s, extension at 72°C/60 s, terminal hold 72°C/10 min. DNA sequencing confirmed correct amplification. The PCR products were purified on agarose gels, using Promega's Wizard SV Gel and PCR Clean‐Up kit. The vector pET28a was linearized by restriction with *Nde*I and *Xho*I (to insert *cubF*, *cub G2*, *cubH*, or *cubI*), or with *Nhe*I and *Xho*I (*cubG2*). Expression plasmids were assembled using the NEBuilder kit (NEB), following the manufacturer's instructions. For gene expression in *A. niger*, the vector pPS01 [29] was used to create plasmids pLD06 (*cubF*), pLD07 (*cubG1*), pLD08 (*cubG2*), pLD09 (*cubH*), pLD10 (*cubI*). These plasmids complement the host's uracil auxotrophy. To integrate inserts, vector pPS01 was cut with restriction enzymes *Spe*I und *Pac*I. Inserts were prepared by PCR and the conditions described above, but using oligonucleotide pairs oLD16/17 (*cubF*), oLD18/19 (*cubG1*), oLD20/21 (*cubG2*), oLD22/23 (*cubH*), and oLD24/25 (*cubI*). Oligonucleotide sequences are given in Table S17.

### 4.5 Heterologous Production of Terpene Synthases

CubF‐CubI were produced as *N*‐terminally polyhistidine‐tagged fusion enzymes in *E. coli* KRX, transformed with pLD01 (CubF), pLD02 (CubG1), pLD03 (CubG2), pLD04 (CubH), or CubI (pLD05), and grown in yeast extract‐tryptone broth, supplemented with 50 µg mL^−1^ kanamycin for selection. The proteins were purified as previously described [46], concentrated on an Amicon Ultra‐15 centrifugal filter (30 kDa cut‐off, Merck), and eluted with reaction buffer (50 mm TRIS, pH 8, 10 mm MgCl_2_). Protein concentrations were determined using the Pierce BCA‐Protein Assay Kit (Thermo) at *λ* = 562 nm.

### 4.6 In Vitro Product Formation Assays

Triplicate reactions were carried out, using 8–25 μm TRIS‐buffered enzymes CubF‐CubI, and 50 μm (2*E*,6*E*)‐FPP or (2*E*)‐GPP (Merck), in a volume of 500 µL and at 30°C, for 60 min. The reactions were extracted with 200 µL *n*‐hexane and centrifuged. A 1 µL aliquot of the samples was analyzed by GC–MS. Reactions which contained heat‐treated enzymes were run as controls.

### 4.7 Transgene Expression in *A*. *niger*

Protoplasts of *A. niger* ATNT16Δ*pyrG*x24 were produced and transformed as described [47]. To select for successful integration of plasmids, cells were inoculated on uracil‐free medium. Full‐length integration of the cDNAs of *cubF*, *cubG1*, *cubG2*, *cubH*, or *cubI* in the transformants was verified by PCR. For the reactions, oligonucleotides oMG108 (forward) and oMG361 (reverse) were used (Table S18), cDNA as template, and 2.5 U *Taq* polymerase (Promega). The above‐mentioned PCR protocol to construct expression plasmids was applied here as well. Verified transformants *A*. *niger* tLD01, tLD02, tLD03, tLD04, and tLD05 (Figure S5) were selected and inoculated in 100 mL liquid *Aspergillus* minimal medium, for 24 h at 30°C, shaking at 140 rpm. After 24 h of incubation, transgene expression was induced by adding 30 µg mL^−1^ doxycycline, and the cultivation proceeded for another 48 h. *A. niger* tPS01 [29] transformed with the empty plasmid pPS01 (for negative control) and non‐induced *A*. *niger* tLD01 ‐ tLD05 served as controls.

### 4.8 Extraction of *Aspergillus* Cultures

To prepare the subsequent chromatographic analysis. The biomass was passed through Miracloth to separate cells and broth. After rinsing the mycelium with water to remove residual medium, it was blotted dry, shock‐frozen in liquid nitrogen, and subsequently ground. *n*‐Hexane was added to the mycelium powder (500 µL per 1 g biomass), shaken, centrifuged, and used for gas chromatography (below).

### 4.9 Solid Phase Microextraction (SPME)

Analysis of volatile terpenes emitted from *P. cubensis* mycelium and fruiting bodies was accomplished by SPME. Vegetative *P. cubensis* mycelium was grown for 7 days under axenic conditions in an agar slant tube containing 2 mL MEP agar. The culture was briefly aerated once a day. After 7 days, the culture was sealed by an airtight septum. To induce mycelial damage, a lysing enzyme solution (mixture of VinoTaste Pro, Novonesis and *Trichoderma harzianum* lysing enzymes, Sigma–Aldrich) was applied onto the mycelium on day 8 [47]. The next day, the SPME fiber (Supelco, portable field sampler, coating 100 μm polydimethylsiloxane) was introduced in the headspace above the mycelium by puncturing the septum to sample volatiles for 24 h. Subsequently, the fiber was retracted, and the adsorbed volatiles subjected to gas chromatography.

For SPME with fruiting bodies, the mushrooms were grown as described [39]. Young fruiting bodies (2 cm tall) were harvested, attached soil particles were carefully removed. Then, the fruiting bodies were transferred to a glass tube, which was sealed. Volatiles were sampled for 3 days using SPME field samplers.

### 4.10 Gas Chromatographic and Mass Spectrometric Analyses

The products of CubF‐CubI assays and SPME headspace collection were analyzed on an ISQ GC–MS‐System (Thermo Fisher Scientific), using a Phenomenex ZB‐5 MS column and inactive guard column (30 + 10 m × 0.25 mm × 0.25 μm), applying published parameters [16]. Beginning at 60°C, the oven temperature was gradually increased by 3 K min^−1^ from 60°C–260°C. Carrier gas flow (helium) was 1 mL min^−1^ with splitless injection. The injector temperature was kept at 220°C. Electron impact ionization was at 70 eV, the ion source was kept at 250°C, the transfer line at 280°C. All spectra where measured in positive ionization mode. To deconvolute the data and to calculate the retention index [48], MassFinder 4.21 (Hochmuth Scientific Consulting, Hamburg, Germany) was used [27]. Using Thermo Fisher Xcalibur v3.1.66.10 software, the GC–MS results were analyzed. Products of CubF‐CubI were identified by comparing their mass spectra and retention indices with databases and literature [24, 25, 26, 27]. We also compared mass spectra and retention times with gas chromatographic runs (applying identical parameters) of pure compounds or, for tentative identification, with essential oils of *Piper cubeba* (Cubeb) berries [49, 50, 51] *Canarium luzonicum* resin (Elemi oil) [52], *Daucus carota* (carrot) seed oil [53], *Angelica archangelica* (Norwegian Angelica) root oil [50], *Meum athamanticum* (Spignel) essential oil [54], *Chamaecyparis obtusa* (Hinoki Cypress) wood oil [55], *Juniperus virginiana* (Red Cedar) wood oil [56], and *Citrus aurantifolia* (Key Lime) essential oil [57].

## Supporting Information

Additional supporting information can be found online in the Supporting Information section.

## Funding

This study was supported by Deutsche Forschungsgemeinschaft (SFB 1127).

## Conflicts of Interest

A. R. C. reports an ownership interest in CaaMTech, LLC.

## Supporting information

Supplementary Material

## Data Availability

The data that supports the findings of this study are available in the supplementary material of this article.
