# Supplementary material for: Specific and Multi‐Product Clade I and Clade IV Sesquiterpene Synthases Contribute to the Psilocybe cubensis Volatilome
Source: Chembiochem. 2026 Apr 21;27(8):e70318. doi: 10.1002/cbic.70318 (PMC13096983; doi:10.1002/cbic.70318)
Supplement: Supplementary file 1 — Supplementary Material [file CBIC-27-e70318-s001.pdf]

Table of Contents

Figure S1. Genetic map of *cubF*, *cubG1, cubG2, cubH, cubI*, and adjacent genes. 2
Figure S2. Phylogenetic tree of basidiomycete clade I and clade IV sesquiterpene synthases 3
Figure S3. qRT-PCR to analyze the expression of *P. cubensis* genes *cubF-cubI*. 4
Figure S4. SDS polyacrylamide gel electrophoresis of purified His_6-tagged terpene synthases............................ 5
Figure S5. PCR analysis of transgene integration in *A. niger* tLD01, tLD02, tLD03, tLD04, tLD05 6
Figure S6. CubF-catalyzed sesquiterpene formation in *Aspergillus niger* tLD01 7
Figure S7. CubG1-catalyzed sesquiterpene formation in *Aspergillus niger* tLD02 8
Figure S8. CubG2-catalyzed sesquiterpene formation in *Aspergillus niger* tLD03 9
Figure S9. CubH-catalyzed sesquiterpene formation in *Aspergillus niger* tLD04 10
Figure S10. CubI-catalyzed sesquiterpene formation in *Aspergillus niger* tLD05 11

Table S1. Sequence similarities of *Psilocybe cubensis* sesquiterpene synthases 12
Table S2. Amino acid sequences of terpene synthases used for phylogenetic analyses 13
Table S3. Sesquiterpenes and -terpenoids produced by CubF *in vitro* 15
Table S4. CubF-catalyzed sesquiterpenes and -terpenoids in *Aspergillus niger* tLD01 15
Table S5. Sesquiterpenes and -terpenoids produced by CubG1 *in vitro* 16
Table S6. Sesquiterpenes and -terpenoids produced by CubG2 *in vitro* 17
Table S7. CubG1-catalyzed terpene formation in *Aspergillus niger* tLD02 18
Table S8. CubG2-catalyzed terpene formation in *Aspergillus niger* tLD03 19
Table S9. Sesquiterpenes and -terpenoids produced by CubH *in vitro* 20
Table S10. Identified products in extracts of *Aspergillus niger* tLD04 20
Table S11. Sesquiterpenes and -terpenoids produced by CubI *in vitro* 21
Table S12. Identified products in extracts of *Aspergillus niger* tLD05 21
Table S13. Headspace analysis of *Psilocybe cubensis* vegetative mycelium.. 22
Table S14. Headspace analysis of *Psilocybe cubensis* fruiting bodies 25
Table S15. Oligonucleotides for qRT-PCR 26
Table S16. Oligonucleotides to construct *Escherichia coli* expression plasmids 26
Table S17. Oligonucleotides to construct *Aspergillus niger* expression plasmids 27
Table S18. Oligonucleotides for diagnostic PCR to verify transgene integration 27

References. 28

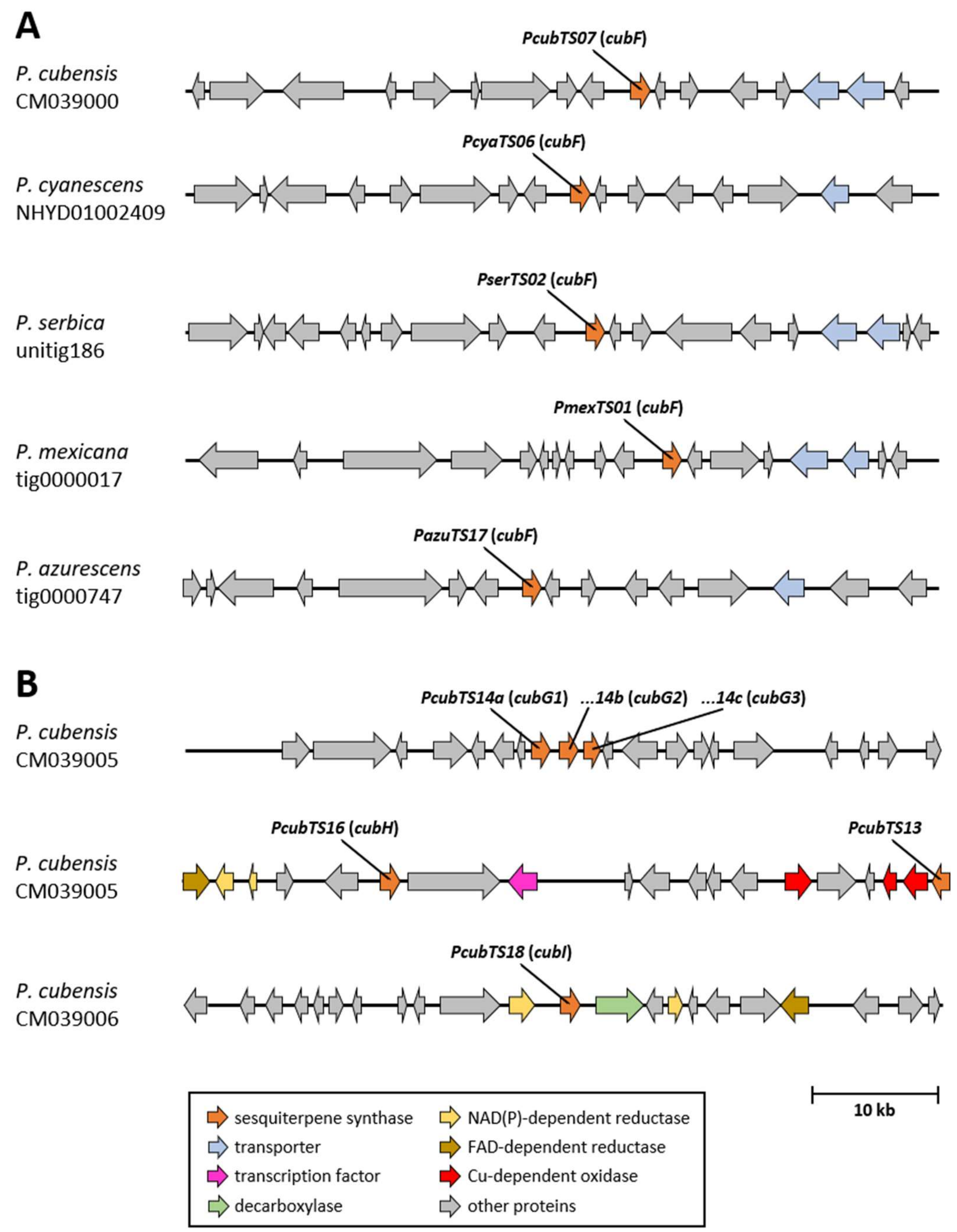

Figure S1. Genetic map of *cubF*, *cubG1, cubG2, cubH,* and *cubI* and adjacent genes.

A) The *cubF* gene was found in *P. cubensis* as well as in the genomes of *P. cyanescens*, *P. serbica*, *P. mexicana* and *P. azurescens*, in a (near-)syntenic region that also encodes one or two major facilitator superfamily-like transporters.

B) The genes *cubG1*, *cubG2*, *cubH*, and *cubI* are solely found in *P. cubensis* and are embedded in potential biosynthetic gene clusters. Please refer to Table S1 for sequence similarities of terpene synthases. The genes encoding *cubH* and *cubI* are located apart from each other in *P. cubensis* and *P. serbica*, but are encoded adjacently in other *Psilocybe* species.

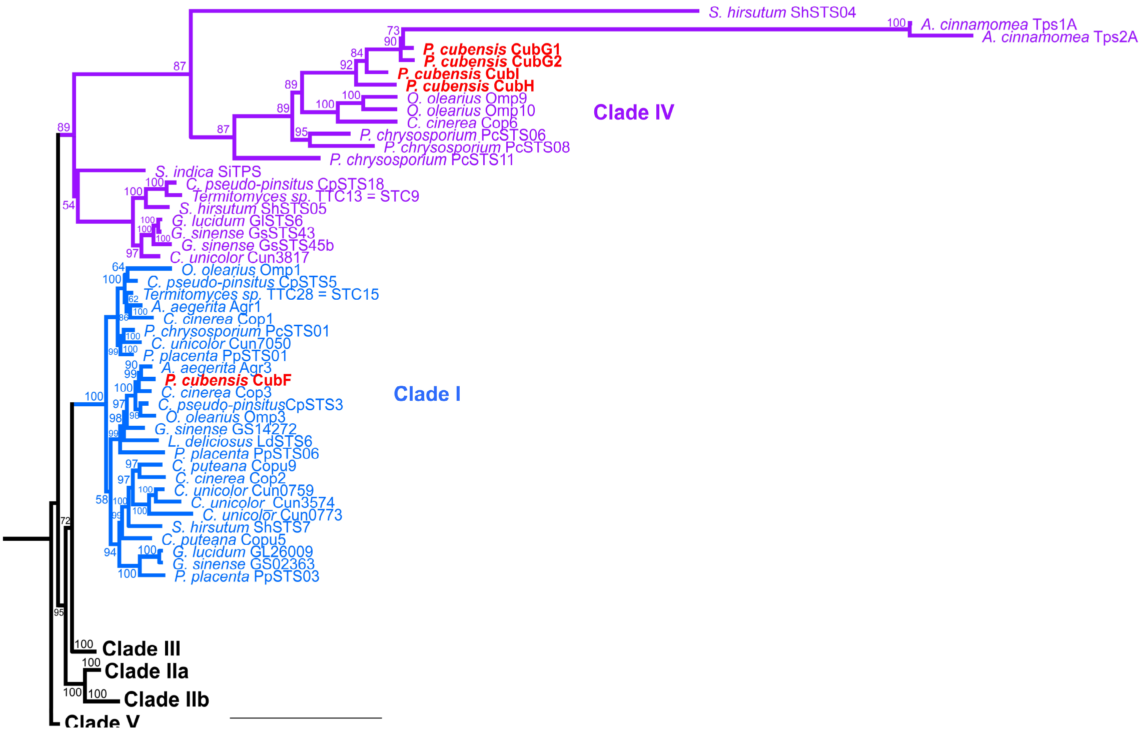

Figure S2. Phylogenetic tree of basidiomycete clade I (blue) and IV (lilac) sesquiterpene synthases and placement of *P. cubensis* synthases CubF, CubG1, CubG2, CubH, and CubI (red) within the cladogram. Details on the enzymes used to build this tree are provided in Table S2. Bootstrap values are indicated at each node. For clarity, clades IIa, IIb, III, and V were collapsed.

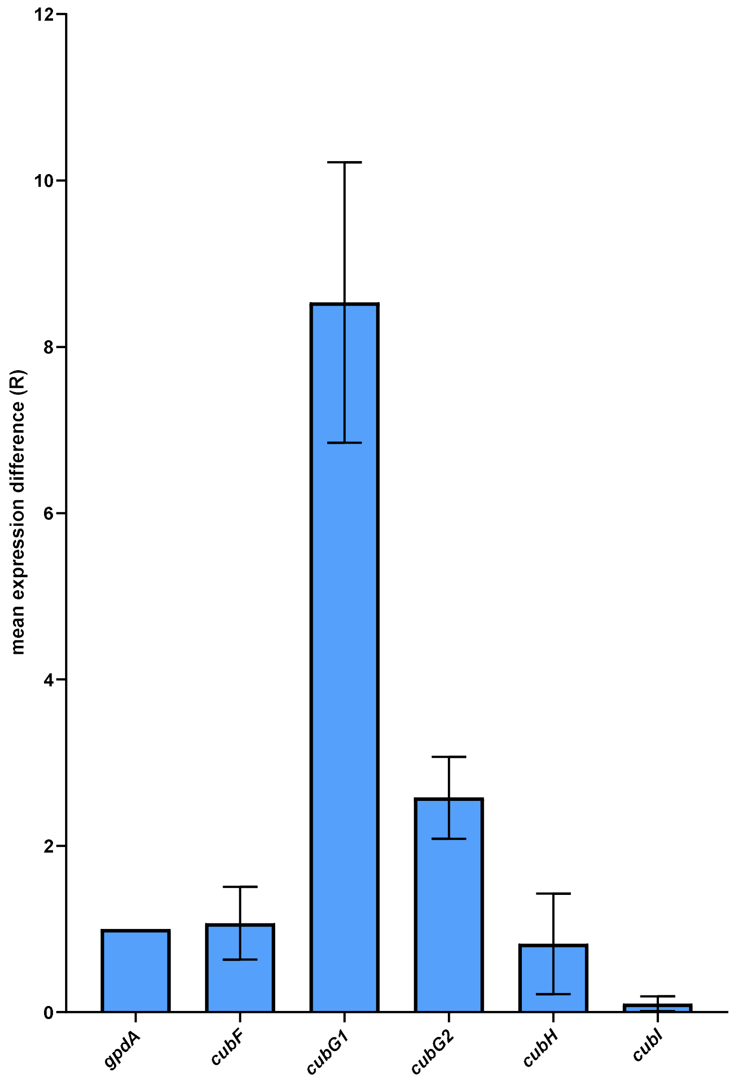

Figure S3. Expression of *Psilocybe cubensis* genes *cubF-cubI*. qRT-PCR was used to compare expression of terpene synthase genes in fruiting bodies with mycelium. The mean expression difference was normalized using the housekeeping gene, *gpdA*, which was set to one. The primers to amplify *cubF* were designed to anneal to up- and downstream exon sequences flanking intron 6. For *cubG1*, the amplicon covers portions of exons 4 and 5, and intron 4, In the case of *cubG2, cubH* and *cubI*, the amplicon includes parts of the third and fourth exon and the third intron. Accurately spliced mRNA leads to amplicons of 102 bp (*cubF*), 110 bp (*cubG1*), 96 bp (*cubG2*), 112 bp (*cubH)* and 144 bp (*cubI*). The bars indicate the standard deviation. Values above and below 1 indicate up-regulation and down-regulation, respectively, in the fruiting body, compared to the mycelium. The highest

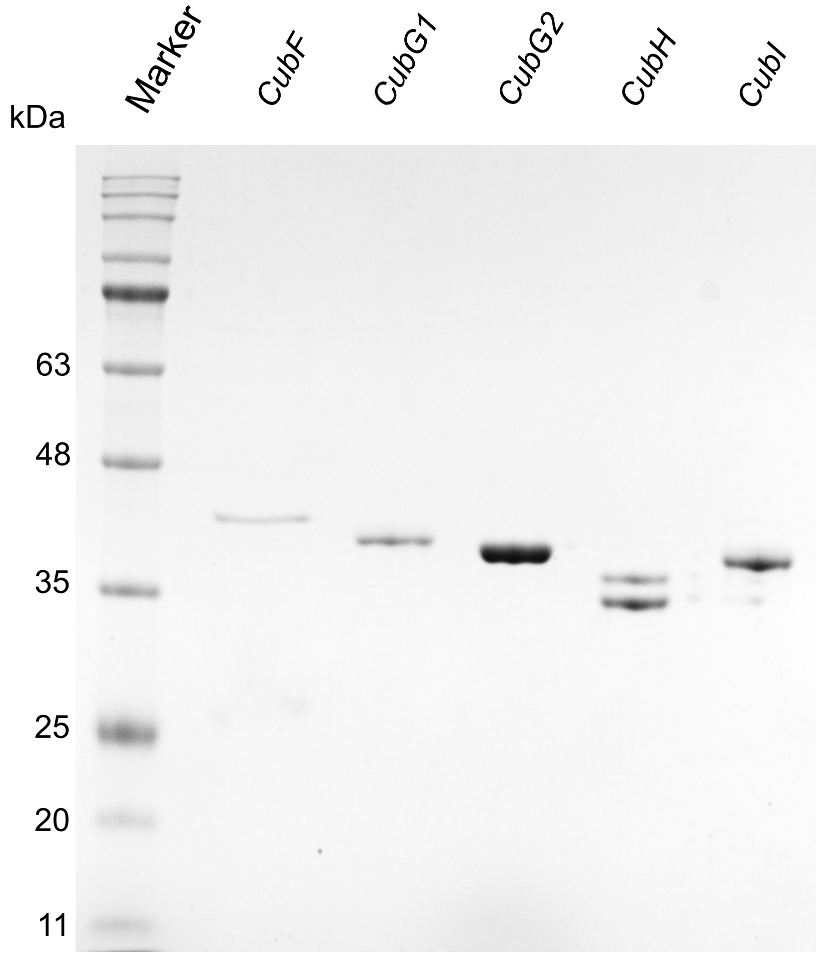

Figure S4. SDS polyacrylamide gel electrophoresis of purified His$_6$-tagged *Psilocybe cubensis* CubF, CubG1, CubG2, CubH, and CubI. The calculated masses of hexahistidin-tagged are 42.2 kDa for CubF, 40.1 kDa for CubG1, 40.2 kDa for CubG2, 38.6 kDa for CubH, 39.0 kDa for CubI. The lower band in the CubH lane reflects an intrinsic instability. Protein standard: Blue Eye prestained marker (Jena Bioscience).

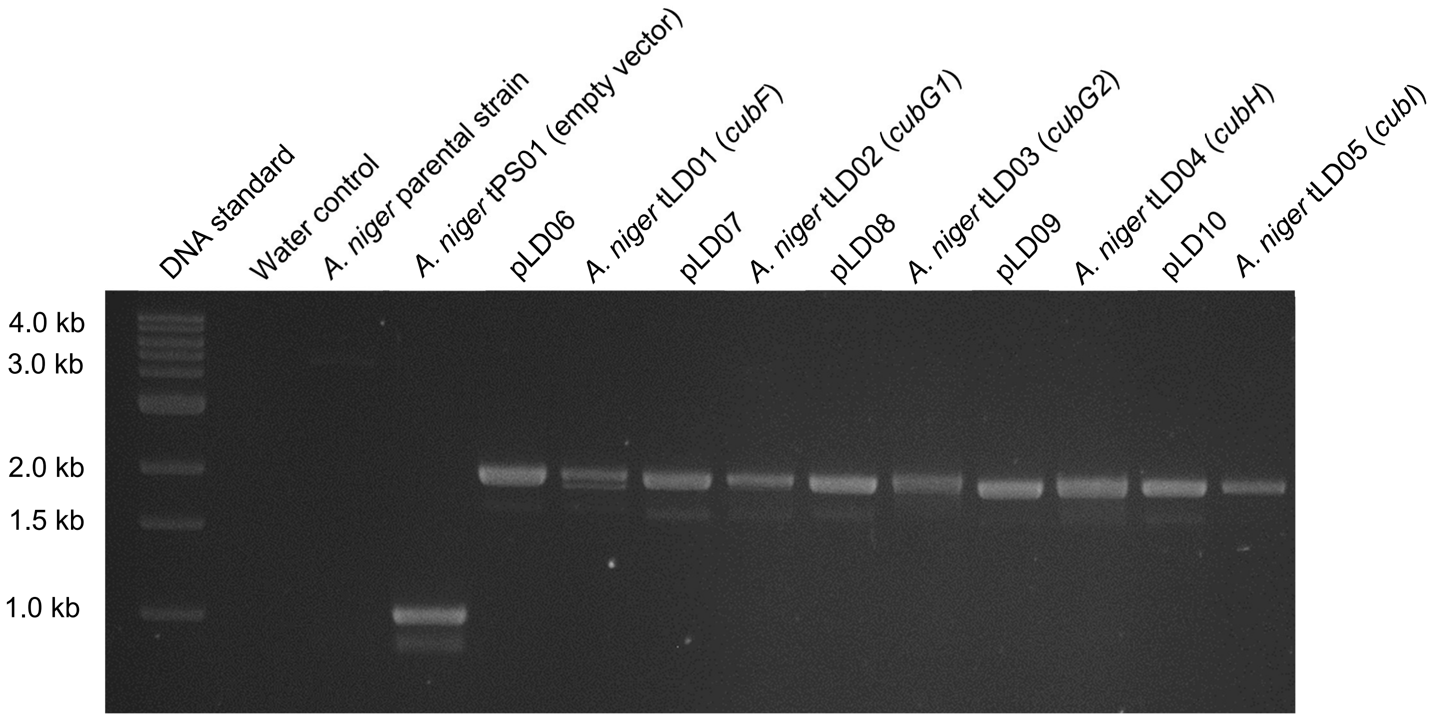

Figure S5. Agarose gel electrophoresis of PCR products to verify integration of the *cubF-cubI* expression constructs in the *Aspergillus niger* genome. The integration yielded producer strains *A. niger* tLD01-tLD05. Negative controls i) with genomic DNA of the untransformed parental strain *A. niger* ATNT16ΔpyrGx24,[28] ii) water control without template DNA, iii) a reaction with empty vector DNA (pPS01),[29] and a positive control (plasmids pLD06-pLD10) are shown as well. DNA standard: 1 kb DNA ladder (NEB). The expected amplicon lengths, covering the complete insert and flanking portions of the vector, are: tPS01: 1006 bp; pLD06/tLD01: 2044 bp; pLD07/tLD02: 1975 bp; pLD08/tLD03: 1987 bp; pLD09/tLD04: 1939 bp; pLD10/tLD05: 1960 bp.

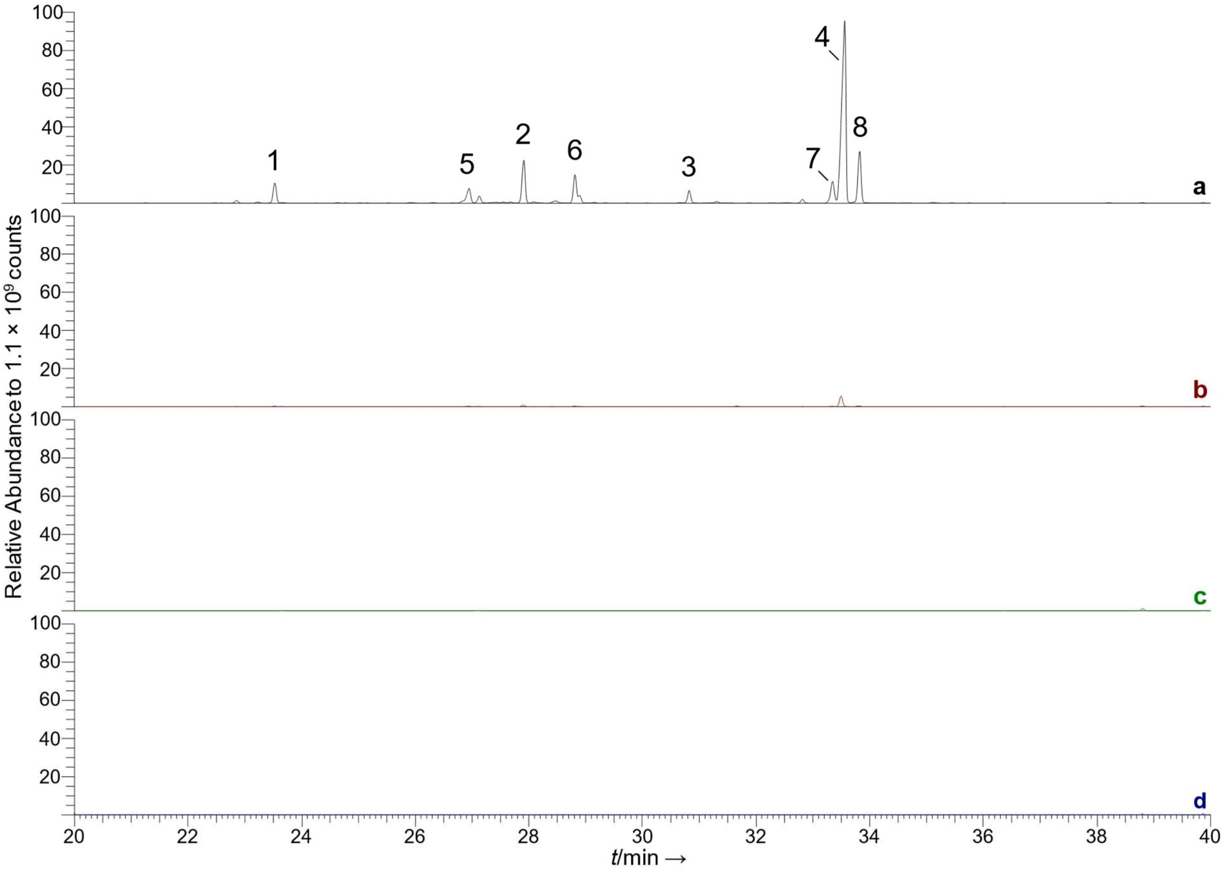

Figure S6. Gas chromatographic analysis of CubF-catalyzed sesquiterpene/-terpenoid formation in *Aspergillus niger* tLD01. Shown are *n*-hexane extracts of (a) a doxycycline-induced and (b) non-induced cultures of *A. niger* tLD01. Chromatograms c and d represent extracts of induced cultures of the empty vector control strain, *A. niger* tPS01 and the parental strain *A. niger* ATNT16ΔpyrGx24.[28] Detected compounds are: β-elemene (**1**), α-muurolene (**2**), germacrene D-4-ol (**3**), α-muurolol (= torreyol) (**4**), γ-muurolene (**5**), δ-cadinene (**6**), τ-muurolol (= *epi*-α-muurolol, **7**), α-cadinol (**8**).

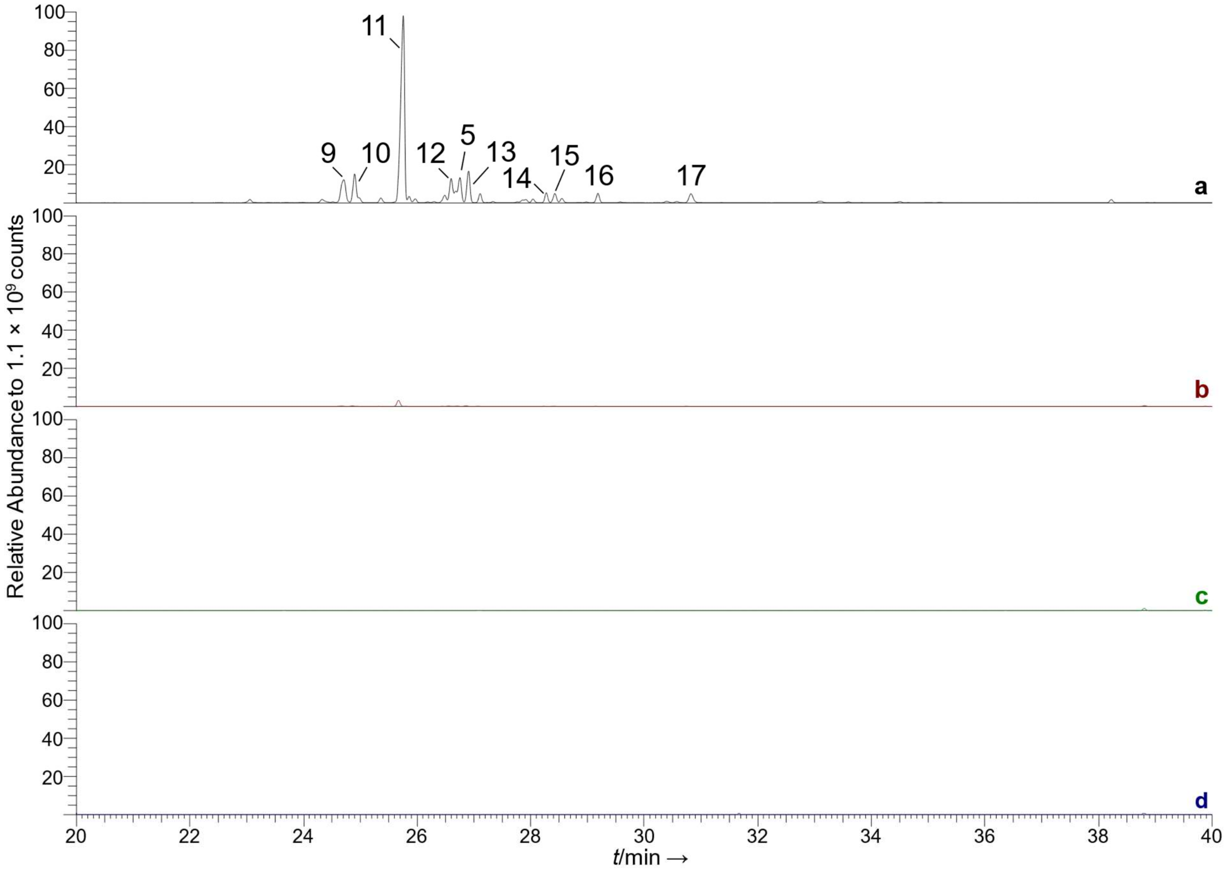

Figure S7. Gas chromatographic analysis of CubG1-catalyzed terpenoid formation in *Aspergillus niger*. Shown are *n*-hexane extracts of (a) doxycycline-induced and (b) non-induced cultures of *A. niger* tLD02. Chromatograms c and d represent extracts of induced cultures of the empty vector control strain, *A. niger* tPS01, and the parental strain *A. niger* ATNT16ΔpyrGx24. Detected compounds are: β-cedrene (**9**), acora-3,5-diene (**10**), *epi*-isozizaene (**11**), β-acoradiene (**12**), γ-muurolene (**5**), 10-*epi*-β-acoradiene (**13**), β-bisabolene (**14**), α-alaskene (=γ-acoradiene) (**15**), (*E*)-γ-bisabolene (**16**), unknown (**17**, 123, 222, RI 1575, base peak, molecular ion, retention index).

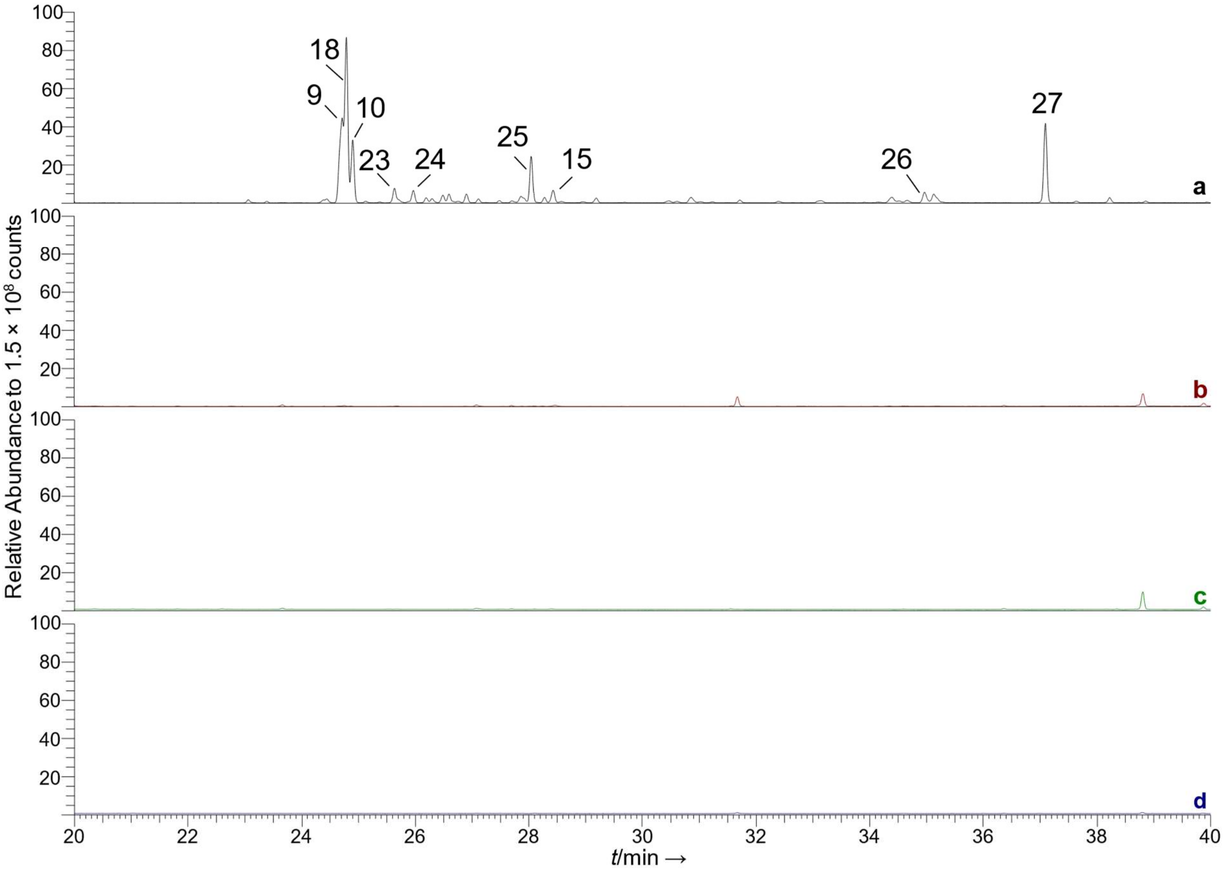

Figure S8. Gas chromatographic analysis of CubG2-catalyzed terpenoid formation in *Aspergillus niger* tLD03. Shown are *n*-hexane extracts of an (a) doxycycline-induced and (b) non-induced culture of *A. niger* tLD03. Chromatograms c and d represent extracts of induced cultures of the empty vector control strain, *A. niger* tPS01, and the parental strain *A. niger* ATNT16ΔpyrGx24. Detected compounds are: β-cedrene (**9**); β-duprezianene (**18**), acora-3,5-diene (**10**), sesquisabinene A (**23**), amorpha-4,11-diene (**24**), (*Z*)-α-bisabolene (**25**), α-alaskene (**15**), unknown (**26**, 151, 222, RI 1686, base peak, molecular ion, retention index), unknown (**27**, 119, 222, RI 1746, base peak, molecular ion, retention index).

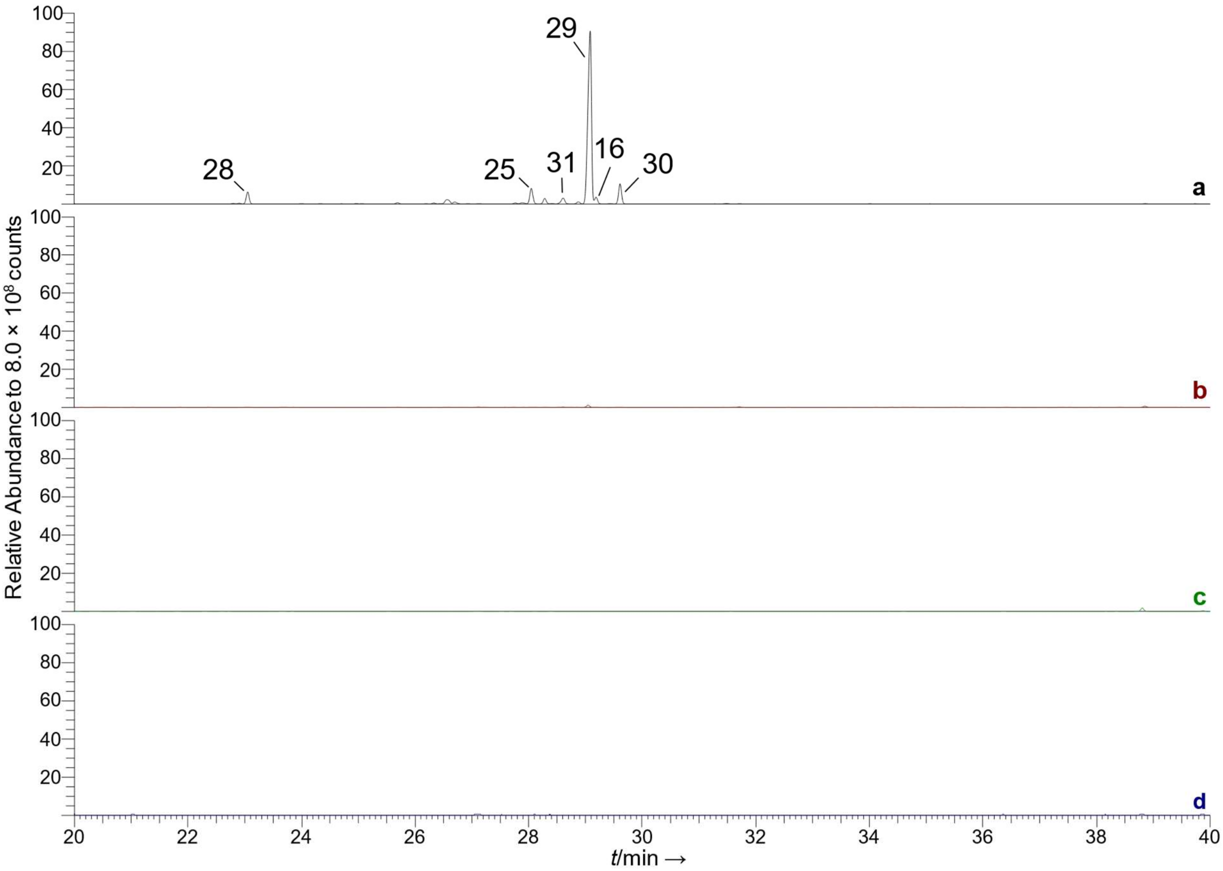

Figure S9. Gas chromatographic analysis of CubH-catalyzed terpenoid formation in *Aspergillus niger* tLD04. Shown are *n*-hexane extracts of an (a) doxycycline-induced and (b) non-induced culture of *A. niger* tLD04. Chromatograms c and d represent extracts of induced cultures of the empty vector control strain, *A. niger* tPS01, and the parental strain *A. niger* ATNT16ΔpyrGx24. Detected compounds are: daucene (**28**), (*Z*)-α-bisabolene (**25**), (*E*)-iso-γ-bisabolene (**31**), dauca-4(11),8-diene (**29**), (*E*)-γ-bisabolene (**16**), unknown (**30**, 93, 204, RI 1544, base peak, molecular ion, retention index).

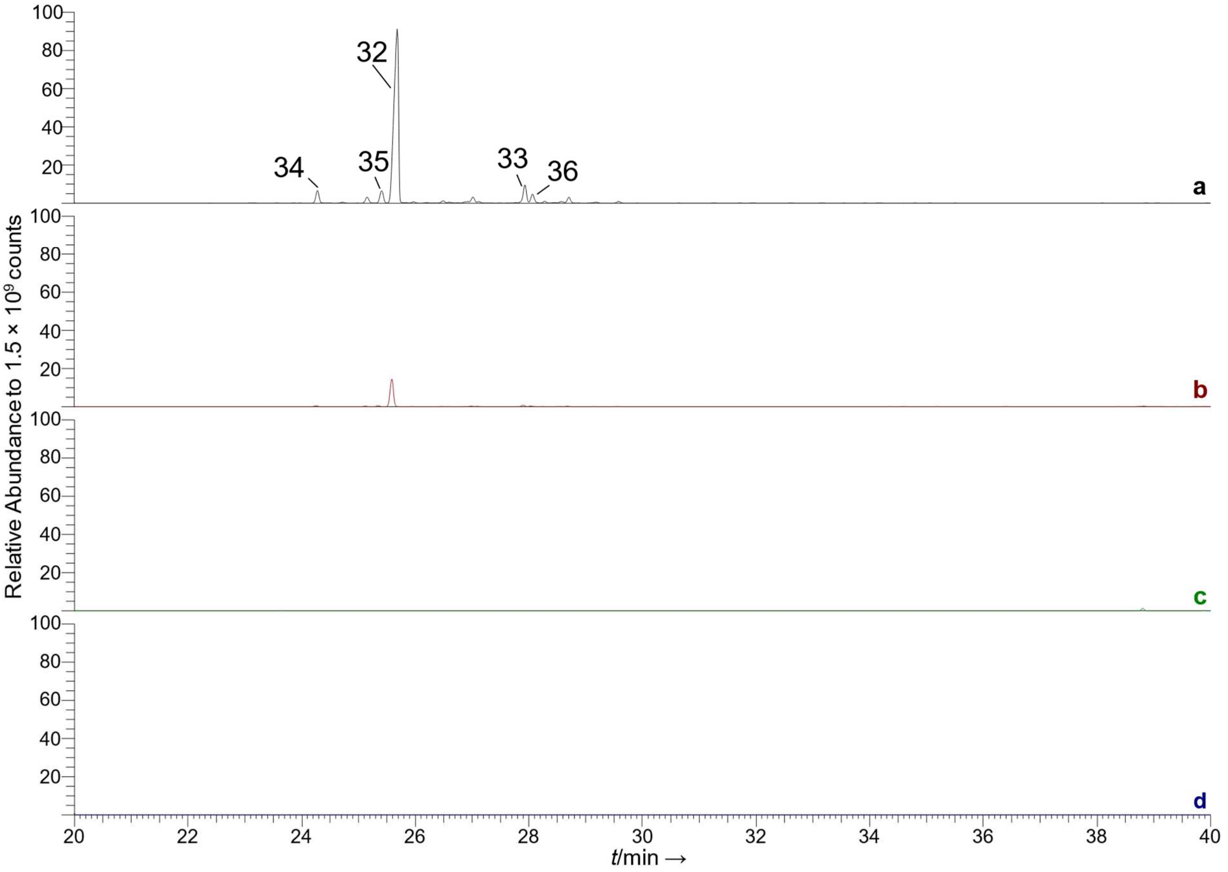

Figure S10. Gas chromatographic analysis of CubI-catalyzed terpenoid formation in *Aspergillus niger* tLD05. Shown are *n*-hexane extracts of an (a) doxycycline-induced and (b) non-induced culture of *A. niger* tLD05. Chromatograms c and d represent extracts of induced cultures of the empty vector control strain, *A. niger* tPS01, and the parental strain *A. niger* ATNT16ΔpyrGx24. Detected compounds are: α-barbatene (**34**), isobazzanene (**35**), β-barbatene (**32**), β-himachalene (**33**), α-chamigrene (**36**).

Table S1. Sequence similarities of *Psilocybe cubensis* sesquiterpene synthases CubF, CubG1, CubG2, CubH, and CubI and enzymes in other *Psilocybe* species. Amino acid sequence identity and similarity (according to Blosum90 analysis) and encoding contigs are indicated.

	P. cubensis	***P. azurescens***	***P. cyanescens***	***P. mexicana***	***P. serbica***
**CubF**		PazuTS17<br>91.9%, 97.2%	PcyaTS6<br>92.3%; 97.4%	PmexTS01<br>81.6%; 92.1%	PserTS02<br>88.1%; 96.3%
contig	CM39000<br>c00556_NODE4317	tig00000747	NHYD01002409	tig00000017	unitig186
**CubG1**		PazuTS09<br>65.8%; 79.1%	PcyaTS05<br>69.2%; 83.8%	PmexTS03a<br>40.1%; 59.9%	PserTS10<br>68.2%; 83.5%
contig	CM39005<br>c00870_NODE6539	tig00000876	NHYD01001919	tig00000088	unitig122
**CubG2**		PazuTS09<br>64.5%; 77.0%	PcyaTS05<br>67.0%; 82.1%	PmexTS03a<br>40.0%; 60.0%	PserTS10<br>66.5%; 81.7%
contig	CM39005<br>c00870_NODE6539	tig00000876	NHYD01001919	tig00000088	unitig122
**CubH**		PazuTS07b<br>62.7%; 77.1%	PcyaTS15b<br>65.9%; 80.6%	PmexTS13b<br>42.1%; 66.9%	PserTS11<br>54.5%; 74.3%
contig	CM39006<br>c01033_NODE9136	tig00000729	NHYD01003422	tig00000291	unitig166
**CubI**	PcubTS18	PazuTS07a<br>67.7%; 83.1%	PcyaTS15a<br>72.4%; 87.3%	PmexTS13a<br>49.4%; 68.9%	PserTS15<br>69.3%; 82.2%
contig	CM39006<br>c01038_NODE9212	tig00000729	NHYD01003422	tig00000291	unitig17

Table S2. Representatives of clade I and clade IV terpene synthases used for the phylogenetic analyses.

Clade	Protein	NCBI, PDB, or JGI ID, GenBank IDs for *cubB-cubE*	Organism	Sequence length (aa)
clade I	Agr1	QGA30877.1	*Agrocybe/Cyclocybe aegerita*	346
	Agr3	QGA30879.1	*Agrocybe/Cyclocybe aegerita*	358
	Cop1	EAU89322.2	*Coprinopsis cinerea okayama7#130*	390
	Cop2	EAU85264.1	*Coprinopsis cinerea okayama7#130*	348
	Cop3	EAU88892.1	*Coprinopsis cinerea okayama7#130*	425
	CpSTS3	BBH51500.1	*Clitopilus pseudo-pinsitus*	346
	CpSTS5	BBH51502.1	*Clitopilus pseudo-pinsitus*	344
	Copu5	XP_007765330.1	*Coniophora puteana RWD-64-598 SS2*	341
	Copu9	7OFL_A	*Coniophora puteana RWD-64-598 SS2*	377
	Cun0759	JGI:Cerun2|378245	*Cerrena unicolor*	376
	Cun0773	JGI:Cerun2|310724	*Cerrena unicolor*	332
	Cun3574	JGI:Cerun2|315667	*Cerrena unicolor*	335
	Cun3817	JGI:Cerun2|380643	*Cerrena unicolor*	399
	Cun7050	JGI:Cerun2|321710	*Cerrena unicolor*	341
	GL26009	JGI:Ganluc1|447316	*Ganoderma lucidum*	346
	GS02363	PIL35634.1	*Ganoderma sinense ZZ0214-1*	346
	GS14272	PIL24516.1	*Ganoderma sinense ZZ0214-1*	359
	LdSTS6	KAH9079282.1	*Lactarius deliciosus*	385
	GME3634	KX281943.1	*Lignosus rhinocerotis*	379
	GME3638	KX281944.1	*Lignosus rhinocerotis*	367
	Omp1	1311|MUStwsD_GLEAN_10001317	*Omphalotus olearius*	333
	Omp3	4636|MUStwsD_GLEAN_10003938	*Omphalotus olearius*	368
	PcSTS01	BCX55496.1	*Phanerodontia chrysosporium*	351
	CubF	PX842263	*Psilocybe cubensis*	352
	PpSTS01	XP_024337827.1	*Postia placenta MAD-698-R-SB12*	338
	PpSTS03	EED82142.1	*Postia placenta Mad-698-R*	365
	PpSTS06	A0A348B782.1	*Postia placenta Mad-698-R*	340

	ShSTS7	XP_007302965.1	*Stereum hirsutum FP-91666 SS1*	343
	TTC28 = STC15	KNZ77998.1	*Termitomyces sp. J132*	342
clade IV	Cop6	EAU89298.1	*Coprinopsis cinerea okayama7#130*	325
	CpSTS17	BBH51514.1	*Clitopilus pseudo-pinsitus*	330
	CpSTS18	BBH51515.1	*Clitopilus pseudo-pinsitus*	393
	Fompi1	EPT01290.1	*Fomitopsis schrenkii*	378
	Gs11330	PIL22360.1	*Ganoderma sinense ZZ0214-1*	311
	Omp9	jgi|Ompol1|3258|MUStwsD_GLEAN_10000543	*Omphalotus olearius*	351
	Omp10	jgi|Ompol1|3981|MUStwsD_GLEAN_10000292	*Omphalotus olearius*	304
	PcSTS06	BCX55500.1	*Phanerodontia chrysosporium*	336
	PcSTS08	BCX55502.1	*Phanerodontia chrysosporium*	338
	PcSTS11	BCX55504.1	*Phanerodontia chrysosporium*	315
	CubG1	PX842264	*Psilocybe cubensis*	326
	CubG2	PX842265	*Psilocybe cubensis*	329
	CubH	PX842266	*Psilocybe cubensis*	316
	CubI	PX842267	*Psilocybe cubensis*	323
	ShSTS01	XP_007306912.1	*Stereum hirsutum FP-91666 SS1*	386
	ShSTS05	XP_007309315.1	*Stereum hirsutum FP-91666 SS1*	350
	SiTPS	CAG7846754.1	*Serendipita indica DSM 11827*	515

Table S3. Products of *in vitro* assays with *Psilocybe cubensis* CubF and (2*E*,6*E*)-FPP as substrate.

Compound	t_R (min)	Integral	Integral (% of total area)	Retention Index (observed)	Retention Index (Adams)[24]	Retention Index (NIST)[25]	Present in/ tentatively identified by comparison with
β-Elemene (**1**)	23.54	1.53×10^6	3.23	1392	1389	1391	*Piper cubeba* oil,[49-51] Elemi oil,[52] *Angelica archangelica* root oil[50]
α-Muurolene (**2**)	27.92	4.56×10^6	12.3	1500	1500	1499	*Piper cubeba* oil, Elemi oil; *Angelica archangelica* root oil, *Chamaecyparis obtusa* wood oil[55]
Germacrene D-4-ol (**3**)	30.85	4.89×10^6	8.51	1575	1574	1574	*Piper cubeba* oil, Elemi oil
α-Muurolol (= Torreyol, **4**)	33.61	1.37×10^7	72.74	1649	1644	1645	*Piper cubeba* oil, Elemi oil, *Angelica archangelica* root oil, *Chamaecyparis obtusa* wood oil

Table S4. Products in extracts of *Aspergillus niger* tLD01. Expression of *cubF* in this transgenic strain was induced by 30 µg mL^{-1} doxycycline.

Compound	t_R (min)	Integral	Integral (% of total area)	Retention Index (observed)	Retention Index (Adams)[24]	Retention Index (NIST)[25]*	Present in/ tentatively identified by comparison with
β-Elemene (**1**)	23.56	9.31×10^7	2.92	1393	1389	1391	*Piper cubeba* oil,[49-51] Elemi oil,[52] *Angelica archangelica* root oil[50]
γ-Muurolene (**5**)	26.99	1.28×10^8	3.11	1478	1478	1477	*Piper cubeba* oil, *Angelica archangelica* root oil, *Chamaecyparis obtusa* wood oil[55]
α-Muurolene (**2**)	27.95	2.85×10^8	9.17	1502	1500	1499	*Piper cubeba* oil, Elemi oil, *Angelica archangelica* root oil, *Chamaecyparis obtusa* wood oil
δ-Cadinene (**6**)	28.86	2.20×10^8	5.74	1525	1522	1524	*Piper cubeba* oil, *Angelica archangelica* root oil
Germacrene D-4-ol (**3**)	30.89	7.12×10^7	2.3	1577	1574	1574	*Piper cubeba* oil

τ-Muurolol (=*epi*-α-Muurolol, **7**)	33.42	1.44×10^8	4.24	1644	1640	1642	*Angelica archangelica* root oil, *Chamaecyparis obtusa* wood oil
α-Muurolol (= Torreyol, **4**)	33.59	1.76×10^9	57.88	1649	1644	1645	*Piper cubeba* oil, *Chamaecyparis obtusa* wood oil
α-Cadinol (**8**)	33.89	3.38×10^8	10.73	1657	1652	1653	*Chamaecyparis obtusa* wood oil

Table S5. Products of *in vitro* assays with *Psilocybe cubensis* CubG1 and (2*E*,6*E*)-FPP as substrate.

Compound	t_R (min)	Integral	Integral (% of total area of sesquiterpenes)	Retention Index (observed)	Retention Index (Adams)[24]	Retention Index (NIST)[25]	Present in/ tentatively identified by comparison with
β-Cedrene (**9**)*	25.25	6.32×10^7	4.39	1422	1419	1421	original reference
Acora-3,5-diene (**10**)	25.42	6.83×10^7	4.34	1426	1421	-	*Juniperus virginiana* wood oil[56]
epi-Isozizaene (**11**)*	26.25	9.17×10^8	67.38	1447	1443	-	original reference
β-Acoradiene (**12**)	27.12	1.18×10^8	5.88	1468	1469	1468	*Juniperus virginiana* wood oil
γ-Muurolene (**5**)	27.27	5.01×10^7	4.06	1472	1478	1477	*Piper cubeba* oil,[49-51] *Angelica archangelica* root oil,[50] *Chamaecyparis obtusa* wood oil[55]
10-*epi*-β-Acoradiene (**13**)	27.42	7.95×10^7	5.86	1476	1474	1475	-

* retention index and mass spectra identical with those of reference compound

Table S6. Products of *in vitro* assays with *Psilocybe cubensis* CubG2 and (2*E*,6*E*)-FPP as substrate.

Compound	t_R (min)	Integral	Integral (% of total area)	Retention Index (observed)	Retention Index (Adams)[24]	Retention Index (NIST)[25]	Present in/ tentatively identified by comparison with
β-Cedrene (**9**)*	24.59	2.38×10^7	4.28	1419	1419	1421	original reference
β-Duprezianene (**18**)	24.67	7.72×10^7	13.89	1421	1421	1421	-
Acora-3,5-diene (**10**)	24.78	2.38×10^7	4.95	1424	1421[27]	-	*Juniperus virginiana* wood oil[56]
unknown (**19**, 119, 204, RI 1543)**	29.52	2.34×10^7	4.21	1543	-	-	-
unknown (**20**, 119, 204, RI 1572)**	30.63	1.98×10^7	3.56	1572	-	-	-
Acorenol (**21**)	34.16	2.58×10^8	46.29	1666	1667[27]	-	-
unknown (**22**, 119, 222, RI 1681)	34.73	7.07×10^7	12.7	1681	-	-	-

* retention index and mass spectra identical with those of reference compound
** base peak, molecular ion, retention index

Table S7. Sesquiterpenes in extracts of *Aspergillus niger* tLD02. Expression of *cubG1* was induced with 30 µg mL^{-1} doxycycline.

Compound	t_R (min)	Integral	Integral (% of total area of sesquiterpenes)	Retention Index (observed)	Retention Index (Adams)[24]	Retention Index (NIST)[25]*	Present in/ tentatively identified by comparison with
β-Cedrene (**9**)*	24.71	7.82×10^8	7.47	1421	1419	1421	original reference
Acora-3,5-diene (**10**)	24.90	6.49×10^8	6.21	1426	1421	-	*Juniperus virginiana* wood oil[56]
epi-Isozizaene (**11**)*	25.75	5.21×10^9	49.76	1447	1443	-	original reference
β-Acoradiene (**12**)	26.60	5.41×10^8	5.08	1468	1469	1468	*Juniperus virginiana* wood oil
γ-Muurolene (**5**)	26.75	5.13×10^8	4.90	1472	1478	1477	*Piper cubeba* oil,[49-51] *Angelica archangelica* root oil[50]
10-*epi*-β-Acoradiene (**13**)	26.90	5.13×10^8	6.40	1476	1474	1475	-
β-Bisabolene (**14**)	28.28	2.10×10^8	2.00	1510	1511	1509	*Piper cubeba* oil,[49-51] *Angelica archangelica* root oil[50]
α-Alaskene (=γ-Acoradiene, **15**)	28.43	1.86×10^8	1.78	1514	1512	1514	*Juniperus virginiana* wood oil
(*E*)-γ-Bisabolene (**16**)	29.19	2.12×10^8	2.03	1533	1529	1533	*Citrus aurantifolia* oil[57]
unknown (**17**, 123, 222, RI 1575)**	30.82	2.69×10^8	2.57	1575	-	-	-

* retention index and mass spectra identical with those of reference compound
** base peak, molecular ion, retention index

Table S8. Sesquiterpenes in extracts of *Aspergillus niger* tLD03. Expression of *cubG2* was induced with 30 µg mL^{-1} doxycycline.

Compound	t_R (min)	Integral	Integral (% of total area)	Retention Index (observed)	Retention Index (Adams)[24]	Retention Index (NIST)[25]*	Present in/ tentatively identified by comparison with
β-Cedrene (**9**)*	24.71	2.95×10^{8}	18.72	1421	1419	1421	original reference
β-Duprezianene (**18**)	24.79	5.19×10^{8}	26.63	1423	1424	1423	-
Acora-3,5-diene(**10**)	24.90	1.76×10^{8}	11.38	1426	1421	-	*Juniperus virginiana* wood oil[56]
Sesquisabinene A (**23**)	25.64	4.60×10^{7}	2.47	1444	1435[27]	1444	-
Amorpha-4,11-diene (**24**)	25.97	3.82×10^{7}	2.05	1452	1449	1457	-
(*Z*)-α-Bisabolene (**25**)	28.04	1.29×10^{8}	6.91	1504	1506	1504	*Citrus aurantifolia* oil[57]
α-Alaskene (**15**)	28.43	3.95×10^{7}	2.12	1514	1512	1515	*Juniperus virginiana* wood oil
unknown (**26**,151, 222, RI 1686)**	34.97	4.00×10^{7}	2.07	1686	1687	-	-
unknown (**27**,119, 222, RI 1746)**	37.09	2.49×10^{8}	15.2	1746	-	-	-

* retention index and mass spectra identical with those of reference compound
** base peak, molecular ion, retention index

Table S9. Products of *in vitro* assays with *Psilocybe cubensis* CubH and (2*E*,6*E*)-FPP as substrate.

Compound	t_R (min)	Integral	Integral (% of total area)	Retention Index (observed)	Retention Index (Adams)[24]	Retention Index (NIST)[25]	Present in/ tentatively identified by comparison with
Daucene (**28**)	23.58	2.58×10^7	2.06	1382	1380	1381	*Daucus carota* seed oil[53]
(*Z*)-α-Bisabolene (**25**)	28.54	3.89×10^7	3.12	1503	1506	1504	*Citrus aurantifolia* oil[57]
Dauca-4(11),8-diene (**29**)	29.58	1.04×10^9	83.09	1530	1530	1531	*Daucus carota* seed oil
unknown (**30**, 93, 204, RI 1544)*	30.11	7.64×10^7	6.26	1544	-	-	-

*base peak, molecular ion, retention index

Table S10. Identified product in extracts of *Aspergillus niger* tLD04. Expression of *cubH* in this transgenic strain was induced with 30 µg mL^{-1} doxycycline.

Compound	t_R (min)	Integral	Integral (% of total area)	Retention Index (observed)	Retention Index (Adams)[24]	Retention Index (NIST)[25]*	Present in/ tentatively identified by comparison with
Daucene (**28**)	23.05	1.82×10^8	4.23	1381	1380	1380	*Daucus carota* seed oil[53]
(*Z*)-α-Bisabolene (**25**)	28.05	2.41×10^8	5.49	1504	1506	1504	*Citrus aurantifolia* oil[57]
unknown (**31,** 119, 204, RI 1518)	28.61	1.07×10^8	2.52	1518	-	-	-
Dauca-4(11),8-diene (**29**)	29.08	3.24×10^9	72.79	1531	1530	1532	*Daucus carota* seed oil
(*E*)-γ-Bisabolene (**16**)	29.19	8.90×10^7	2.33	1533	1529	1533	*Citrus aurantifolia* oil
unknown (**30**, 93, 204, RI 1544)*	29.61	3.17×10^8	7.12	1544	-	-	-

*base peak, molecular ion, retention index

Table S11. Identified products in *in vitro* assays with *Psilocybe cubensis* CubI and (2*E*,6*E*)-FPP as substrate.

Compound	t_R (min)	Integral	Integral (% of total area)	Retention Index (observed)	Retention Index (Adams)[24]	Retention Index (NIST)[25]	Present in/ tentatively identified by comparison with
β-Barbatene (**32**)	26.16	9.81×10^8	90.89	1444	1440	1446	*Angelica archangelica* root oil,[50] *Meum athamanticum* root oil[54]
β-Himachalene (**33**)*	28.46	3.05×10^7	2.55	1501	1504	1500	original reference, *Meum athamanticum* root oil

* retention index and mass spectra identical with those of reference compound

Table S12. Identified product in extracts of *Aspergillus niger* tLD05. Expression of *cubI* in this transgenic strain was induced with 30 µg mL^{-1} doxycycline.

Compound	t_R (min)	Integral	Integral (% of total area)	Retention Index (observed)	Retention Index (Adams)[24]	Retention Index (NIST)[25]*	Present in/ tentatively identified by comparison with
α-Barbatene (**34**)	24.28	3.85×10^8	3.91	1411	1407	1416	*Meum athamanticum* root oil[54]
Isobazzanene (**35**)	25.41	4.07×10^8	4.11	1439	1436	-	*Juniperus virginiana* wood oil,[56] *Meum athamanticum* root oil
β-Barbatene (**32**)	25.68	7.60×10^9	76.69	1445	1440	1446	*Angelica archangelica* root oil,[50] *Meum athamanticum* root oil
β-Himachalene (**33**)*	27.93	5.92×10^8	5.52	1501	1504	1500	original reference, *Meum athamanticum* root oil
α-Chamigrene (**36**)	28.06	2.76×10^8	2.48	1504	1507	1507	*Juniperus virginiana* wood oil, *Meum athamanticum* root oil

* retention index and mass spectra identical with those of reference compound

Table S13. Headspace analysis by solid phase microextraction of *Psilocybe cubensis* mycelium (untreated or treated with lysing enyzmes).

Compounds detected in the headspace of untreated vegetative mycelium

Compound	t_R (min)	Integral	Integral (% of total area of sesquiterpenes)*	Retention Index (observed)	Retention Index (database)[24]	Retention Index (NIST)[25]	Present in/ tentatively identified by comparison with	Synthesizing Enzyme
Sterpurene (**37**)	21.95		trace	1352	1351	-	-	CubD, CubE
Neotrifaradiene (**38**)	22.24		trace	1359	1365	-	-	?
unknown (40, 204, RI 1404)**	24.14		trace	1404	-	-	-	?
α-Gurjunene	24.41		trace	1411	1409	1409	-	?
β-Cedrene (**9**)***	24.86		trace	1422	1419	1421	original reference	CubG1, CubG2
β-Duprezianene (**18**)	24.91		trace	1423	1423	1421	-	CubG2
Acora-3,5-diene (**10**)	25.02		trace	1426	1421	1423	*Juniperus virginiana* wood oil[56]	CubG1, CubG2
β-Copaene	25.20		trace	1430	1430	1432	*Angelica archangelica* root oil,[50]	CubA

* compound concentration too low for quantification
** base peak, molecular ion, retention index
*** retention index and mass spectra identical with those of reference compound

Compounds detected in the headspace of vegetative mycelium following a 48 h incubation with lysing enzymes.

Compound	t_R (min)	Integral	Integral (% of total area of sesquiterpenes)	Retention Index (observed)	Retention Index (database)[24]	Retention Index (NIST)[25]	Present in/ tentatively identified by comparison with	Synthesizing Enzyme
unknown (161, 204, RI 1271)*	18.57	2.20×10^8	6.77	1271	-	-	-	CubD, CubE
Brasila-1,10-diene	20.01		trace	1305	1307	-	-	?
unknown (94, 204, RI 1313)*	20.32	4.00×10^8	12.48	1313	-	-	-	CubD, CubE
unknown (189, 204, RI 1320)*	20.63	9.33×10^7	2.91	1320	-	-	-	CubE
Silphiperfol-5-ene	20.91	5.50×10^7	1.72	1327	1326	1328	-	?
δ-Elemene	21.39	6.44×10^6	0.20	1339	1335	1338	-	?
unknown (108, 204, RI 1347)*	21.74	6.38×10^7	1.99	1347	-	-	-	CubD,CubE
Sterpurene (**37**)	21.96	1.14×10^9	35.59	1352	1351	-	-	CubD, CubE
African-1-ene	22.07	1.21×10^7	0.38	1355	1356	-	-	CubD, CubE
Neotrifaradiene (**38**)	22.24	4.19×10^8	13.07	1359	1365	-	-	CubD, CubE
unknown (131, 204, RI 1364)*	22.45	8.63×10^6	0.27	1364	-	-	-	?
unknown (161, 204, RI 1370)*	22.71	4.42×10^7	1.38	1370	-	-	-	?
Silphiperfol-6-ene	23.01	1.22×10^8	3.82	1377	1377	1378	-	?
unknown (148, 204, RI 1386)*	23.39	1.17×10^8	3.65	1386	-	-	-	CubD,CubE
β-Elemene (**1**)	23.68	8.86×10^7	2.76	1393	1389	1391	*Piper cubeba* oil,[49-51] Elemi oil[52]	CubA, CubC, CubF
(*E*)-β-Caryophyllene (**39**)	24.81	7.99×10^7	2.49	1421	1420	1419	original reference	CubC
β-Duprezianene (**18**)	24.90		trace	1423	1419	1423	-	CubG1, CubG2
β-Cedrene (**9**)	24.93		trace	1421	1419	1421	original reference	CubG1, CubG2

Acora-3,5-diene (**10**)	25.03		trace	1426	1421	1423	*Juniperus virginiana* wood oil[56]	CubG1, CubG2
Dauca-3,8-diene	25.08		trace	1428	1428	-		CubH
β-Copaene	25.21		trace	1431	1430	1430	*Piper cubeba* oil[49-51]	CubA
unknown (131, 202, RI 1442)	25.67	3.05×10^8	9.52	1442	1440	-	-	?
α-Humulene	26.18	9.38×10^6	0.29	1455	1454	1454	original reference	CubC

*base peak, molecular ion, retention index

Table S14. Headspeace analysis by solid phase microextraction of *Psilocybe cubensis* fruiting bodies (untreated or treated with lysing enyzmes). Sesquiterpenes with peak areas below 1% of the total area under the curves are not listed.

Compounds detected above untreated fruiting bodies.

Compound	t_R (min)	Integral	Integral (% of total area of sesquiterpenes)	Retention Index (observed)	Retention Index (database)[24]	Retention Index (NIST)[25]	Present in/ tentatively identified by comparison with	Synthesizing Enzyme
Daucene (**28**)	22.95	1.94×10^6	8.01	1381	1380	1380	*Daucus carota* seed oil[53]	CubH
(*E*)-β-Caryophyllene (**39**)	24.55	3.18×10^6	12.65	1420	1420	1419	original reference	CubC
epi-Isozizaene (**11**)	25.58	1.07×10^6	4,39	1446	1443	-	original reference	CubG1
Dauca-5,8-diene	26.56	3.80×10^5	1.56	1470	1471	1471	*Daucus carota* seed oil	CubH?
(*Z*)-α-Bisabolene (**25**)	27.88	3.60×10^6	14.83	1503	1506	1504	*Citrus aurantifolia* oil	CubG2
Dauca-4(11),8-diene (**29**)	28.90	1.41×10^7	58.09	1529	1530	1531	*Daucus carota* seed oil	CubH

Compounds detected above fruiting bodies following an 48 h incubation with lysing enzymes.

Compound	t_R (min)	Integral	Integral (% of total area of sesquiterpenes)	Retention Index (observed)	Retention Index (database)[24]	Retention Index (NIST)[25]	Present in/ tentatively identified by comparison with	Synthesizing Enzyme
Daucene (**28**)	22.95	trace	-	1381	1380	1381	*Daucus carota* seed oil[53]	CubH
β-Copaene	24.95	trace	-	1430	1430	1430	*Angelica archangelica* root oil[50]	CubA
β-Barbatene (**32**)	25.48	1.05×10^7	70.6	1443	1440	1446	*Angelica archangelica* root oil	CubI
Dauca-4(11),8-diene (**29**)	28.90	4.35×10^7	29.4	1529	1530	1531	*Daucus carota* seed oil	CubH

Table S15. Oligonucleotides used for qRT-PCR. Primer efficiency, the linear correlation coefficient (R^2) and the expected amplicon size are indicated for the respective primer pairs.

Name	Sequence (5´→ 3´)	Target	Efficiency (R^2)	Amplicon size (bp) gDNA	cDNA
oMG388	GTGTCAACAACAACATCATTCC	*gpdA*	88% (0.9958)	208	133
oMG389	AGATCAACGACAGAGACATCG				
oKFW18	GTCATGGAACCCAGAAATTGACG	*cubF*	94% (0.9995)	169	102
oLD03	TAACGGCGTGTCTGGAAGC				
oSSCH132	GCCCTGCAGTTTACAAAAGC	*cubG1*	95% (0.9988)	142	80
oSSCH133	CGTGCCAAGGTAAAGTGAAAACC				
oKFW20	GACCACCTATATATCTGTCATGGAGG	*cubG2*	93% (0.9997)	155	96
oLD09	GTTTCCCCACCGAGAAATTCTTTG				
oKFW21	GAGCTGAATATTGAACTGCCGG	*cubH*	94% (0.9996)	181	112
oLD12	GTTCCCCACTCTGATATTCTTTGTG				
oKFW22	CGCAGCCTACGGATTCTTC	*cubI*	95% (0.9987)	199	144
oLD15	CTCTCCCCAGCAATATCTTCTTTATAG				

Table S16. Oligonucleotides to construct expression plasmids for use in *Escherichia coli*. Recognition sites for restriction enzymes *Nde*I (red), *Nhe*I (blue), *Xho*I (black) are shown in bold.

Name	Sequence (5´→ 3´)	Target (cDNA)
oLD01	TGCCGCGCGGCAGC**CATATG**ATGACTATTGCTTTGACCCCCG	*cubF*
oLD02	TGGTGGTGGTGGTG**CTCGAG**TCAAACCCTCAATGGCAAC	
oLD04	GCGGCAGCCATATG**GCTAGC**ATGGCCACTGTGCTACCG	*cubG1*
oLD05	CTCAGTGGTGGTGGTGGTGGTG**CTCGAG**TCATAATCCTAGTTCG	
oLD07	TGCCGCGCGGCAGC**CATATG**ATGGCCGCCGTTGTTCTT	*cubG2*
oLD08	TGGTGGTGGTGGTG**CTCGAG**TTAAAGTTCAAGTTCGTTCAGCCG	
oLD10	TGCCGCGCGGCAGC**CATATG**ATGGAAGCAAACCAGGACTACTC	*cubH*
oLD11	TGGTGGTGGTGGTG**CTCGAG**CTAATTCGACCCTTCACGATCTTGG	
oLD13	TGCCGCGCGGCAGC**CATATG**ATGGCGTCCACAGCACTC	*cubF*
oLD14	TGGTGGTGGTGGTG**CTCGAG**TCAAGCGATCCCAAGGTCG	

Table S17. Oligonucleotide primers to construct *Aspergillus niger* expression plasmids. Recognition sites for *SpeI* are shown in green, for *PacI* in red.

Name	Sequence (5´→ 3´)	Target (cDNA)
oLD16	TTCTCATCACAGCACCATG**ACTAGT**ATGACTATTGCTTTGACCCCCG	*cubF*
oLD17	GAAATCACTGCTGCTAG**TTAATTAA**TCAAACCCTCAATGGCAACAAC	
oLD18	CAGCACCATG**ACTAGT**ATGGCTAGCATGGCCACTGTGCTACCG	*cubG1*
oLD19	GAAATCACTGCTGCTAG**TTAATTAA**TCATAATCCTAGTTCGTTTAGGCG	
oLD20	TCATCACAGCACCATG**ACTAGT**ATGATGGCCGCCGTTGTTCTTC	*cubG2*
oLD21	GAAATCACTGCTGCTAG**TTAATTAA**TTAAAGTTCAAGTTCGTTCAGCCG	
oLD22	TCATCACAGCACCATG**ACTAGT**ATGATGGAAGCAAACCAGGACTACTC	*cubH*
oLD23	GAAATCACTGCTGCTAG**TTAATTAA**CTAATTCGACCCTTCACGATCTTGG	
oLD24	TCATCACAGCACCATG**ACTAGT**ATGATGGCGTCCACAGCACTC	*cubI*
oLD25	GAAATCACTGCTGCTAG**TTAATTAA**TCAAGCGATCCCAAGGTCG	

Table S18. Oligonucleotide primers to verify transgene integration by diagnostic PCR.

Name	Sequence (5´→ 3´)	Target
oMG108	GGATGTGCTGCAAGGCGATTAAG	vector pPS01
oMG361	GAATTTTACCAGTGGCCTAGG	vector pPS01

References

[24] R. P. Adams, *Identification of Essential Oil Components by Gas Chromatography/Mass Spectrometry*, Allured Publishing Corp., Carol Stream, IL, **2007**.

[25] National Institute for Standards and Technology, Mass Spectral & Retention Index Library, **2017**.

[26] D. Joulain, W. A. König, *The Atlas of Spectral Data of Sesquiterpene Hydrocarbons*, E. B. Verlag, Hamburg, **1998**.

[27] Massfinder. 4.21 ed., Hochmuth Scientific Consulting, Hamburg, **2010** (MS/RI-library, mainly based on [26] with additional compounds).

[28] E. Geib, F. Baldeweg, M. Doerfer, M. Nett, M. Brock, *Cell Chem. Biol.* **2019**, *26*, 223–234.

[29] P. S. Seibold, C. Lenz, M. Gressler, D. Hoffmeister, *J. Antibiot*. **2020**, *73*, 711–720.

[49] R. Bos, H. J. Woerdenbag, O. Kayser, W. J. Quax, K. Ruslan, Elfami, *J. Essent. Oil Res*. **2007**, 19, 14–17.

[50] J. C. Chalchat, R. P. Garry, *J. Essent. Oil Res*. **1997**, 9, 311−319.

[51] E. Al-Sayed, H. A. Gad, D. M. El-Kersh, *ACS Omega* **2021**, *6*, 25652−25663.

[52] M. A. Villanueva, R. C. Torres, K. H. C. Baser, T. Özek, M. Kürkçüoglu M, *Flavour Frag. J.* **1993**, *8*, 35−37.

[53] F. Perineau, L. Ganou, A. Gaset, *Ind. Aliment. Agric*. **1991**, *108*, 357−366.

[54] W. A. König, A. Rieck, Y. Saritas, I. H. Hardt, K.-H. Kubeczja, *Phytochemistry* **1996**, *42*, 461−464.

[55] B. Shieh, Y. Iizuka, Y. Matsubara, *Agr. Biol. Chem.* **1981**, *45*, 1497−1499.

[56] R. P. Adams, Cedarwood oil - analyses and properties. In: Modern methods of plant analysis new series. Vol. 12. Essential Oils and Waxes. Edits., H. F. Linskens and J. F. Jackson, pp. 159−173, Springer. New York, **1991**.

[57] P. Dugo, A. Cotroneo, I. Bonarcorsi, L. Mondello, *Flav. Fragr. J.*, **1998**, *13*, 93−97.
